# Effectiveness of Digital Interventions for Deficit-Oriented and Asset-Oriented Psychological Outcomes in the Workplace: A Systematic Review and Narrative Synthesis

**DOI:** 10.3390/ejihpe12100102

**Published:** 2022-10-03

**Authors:** Maria Armaou, Evangelia Araviaki, Snigdha Dutta, Stathis Konstantinidis, Holly Blake

**Affiliations:** 1 School of Health Sciences, University of Nottingham, Nottingham NG7 2HA, UK; 2 Independent Researcher, London NW1 9AQ, UK; 3 Cambridge Centre for Teaching and Learning, University of Cambridge, Cambridge CB2 3PT, UK; 4 National Institute for Health Research (NIHR), Nottingham Biomedical Research Centre, Nottingham NG7 2UH, UK

**Keywords:** systematic review, digital interventions, workplace

## Abstract

Background: Digital psychological interventions can target deficit-oriented and asset-oriented psychological outcomes in the workplace. This review examined: (a) the effectiveness of digital interventions for psychological well-being at work, (b) associations with workplace outcomes, and (c) associations between interventions’ effectiveness and their theory-base. Methods: six electronic databases were searched for randomised controlled trials (RCT) and quasi-experimental studies. The methodological quality of studies that used randomisation was conducted with the “Cochrane Collaboration’s Risk of Bias” tool, while the “JBI Critical Appraisal Checklist” was used for non-randomised studies. Studies’ theory-base was evaluated using an adaptation of the “theory coding scheme” (TSC). Due to heterogeneity, narrative synthesis was performed. Results: 51 studies were included in a synthesis describing four clusters of digital interventions: (a) cognitive behavioural therapy, (b) stress-management interventions and workplace well-being promotion, (c) meditation training and mindfulness-based interventions, and (d) self-help interventions. Studies demonstrated a high risk of contamination effects and high attrition bias. Theory-informed interventions demonstrated greater effectiveness. Cognitive behavioural therapy demonstrated the most robust evidence for reducing depression symptoms among healthy employees. With the exception of the Headspace application, there was weak evidence for meditation training apps, while relaxation training was a key component of effective stress-management interventions.

## 1. Introduction

There has been a growing need for workplace interventions as occupational outcomes associated with poor mental well-being have been on the increase in recent years. In particular, absenteeism, presenteeism and turnover have increased in recent years costing UK employers between GBP 42 and GBP 45 bn a year, representing a 16% rise since 2016 [1]. Furthermore, UK Labour Force Survey results show that mental ill-health has risen to account for 51% of all work-related ill health, compared to 2018/19 when it accounted for 44% of all work-related ill-health, [2,3]. A similar trend is manifested by the annual NHS staff survey results, showing that in 2020 44% of their participants reported that they felt unwell due to stress at work, whereas the same metric the year before was 40.3%, and in 2016 it was 36.8% [4,5,6].

Workplace interventions frequently tend to incorporate individual-level psychological interventions. There is evidence supporting the effectiveness of in-person psychological interventions but there is considerable variation in their approaches and their intended outcomes [7,8,9,10,11,12,13]. What often distinguishes interventions, and their intended outcomes, is their approach towards psychological well-being at work. This in turn shapes intervention components and mechanisms. For example, improvement of psychological well-being can be defined both as reducing poor mental health indicators (deficit-oriented outcomes) and as increasing positive mental health (asset-oriented outcomes). A separatist, though, approach on psychological well-being can create further challenges for well-being promotion in organisations. For example, stress prevention interventions focus on modifying risk factors for poor mental well-being at work, whereas stress management tends to target individuals’ coping and stress-management skills before symptoms’ initiation [9,14]. However, this distinction can become less clear at times with secondary prevention becoming over the years increasingly integrated within organisations’ employee well-being programmes [15,16]. A holistic approach to psychological well-being promotion is also reflected in guidelines for mental health prevention at work [12,17,18]. In particular, LaMontagne et al. [12] argued that workplace interventions targeting mental health problems need to adopt an integrated approach focusing on (1) reducing work-related risk factors; (2) promoting the development of positive aspects including worker strengths and positive capacities, and (3) addressing mental health problems.

As a concept psychological well-being not only addresses deficit-oriented psychological outcomes indicating poor mental health but also encompasses asset-oriented psychological parameters (e.g., subjective well-being, autonomy, positive relationships etc.) [19,20,21,22,23,24,25]. For example, previous research also shows that positive cognitive/affective states may play a critical role in the creation of resilient workplaces and employee engagement [26,27,28,29,30,31]. Based on the conservation of resources theory, a generic definition of resources is ‘anything perceived by the individual to help attain his or her goals’ [32]. In recent years, psychological well-being promotion has been associated with the development and interplay of psychosocial resources at multiple levels of analysis within organisations [33,34,35]. Similarly, Schaufeli’s [36] online assessment tool (‘Energy Compass’) balances a negative and positive approach to work-related well-being allowing organisations to assess psychological and social resources at work and understand their impact on employee well-being.

It is evident that theoretical underpinnings of psychological well-being promotion shape intervention focus, effectiveness measures and intervention delivery parameters [11,13,37]. With many organisations, though, adopting for the first time remote or hybrid models of work over the pandemic [38]; and digital tools being increasingly used for the delivery of workplace interventions due to their cost-effectiveness, scalability and promise for anonymity and stigma reduction [39,40,41], it is essential to highlight determinants of their effectiveness.

Recent reviews show that digital interventions can reduce common mental health concerns at work and may improve work performance [23,42]. However, there are numerous issues about digital interventions’ theoretical base, intended outcomes and methods that may obscure the systematic evaluation of their findings. Common problematic areas involve the incompatibility of evidence elicited at the group level to the context of digital interventions, an often weak theoretical base, along with challenges associated with diverse delivery modalities and difficulties with setting up robust controlled studies [43,44]. A characteristic example of the theoretical disparity of the field is the variations among digitally delivered interventions for perceived stress among nurses that are ranging from large eMental health programmes to standalone stress-management interventions and eHealth training modules [20,45,46,47,48]. Furthermore, there is significantly less rigorous research on secondary outcomes of digital psychological interventions at work addressing occupational outcomes [13,49]. For this reason, this systematic review will report on the effectiveness of digital psychological interventions at work and assess its association with the interventions’ theoretical underpinnings and explore their associations with occupational outcomes. The review objectives as reported in the review’s protocol [50] were:

(1) To describe the effectiveness of digital interventions for psychological well-being including: (i) improvement of asset-oriented psychological outcomes at work; (ii) the prevention/management of poor mental well-being in the workplace.
(2) To explore the relationship between interventions’ effectiveness and their theoretical base.
(3) To explore the effects of digital interventions on occupational outcomes as secondary intervention outcomes.

## 2. Materials and Methods

### 2.1. Study Design

The review is reported following the Preferred Reporting Items for Systematic Reviews and Meta-Analyses (PRISMA) reporting guidelines. The study protocol was prospectively registered with PROSPERO (CRD42019142428) and published [50].

### 2.2. Eligibility Criteria

#### 2.2.1. Inclusion Criteria

The study’s search strategy was based on PICO-elements (Interventions, Comparators, Outcomes) that reflect the screening criteria against which studies were screened.

(a) Participants

Included studies’ participants needed to be current ‘employees’ including working-age adults and those over 65 years that were still in a contracted role within their organisations.

(b) Interventions

For studies to be included, they needed to report the results of workplace interventions. Interventions could be delivered via any digital method and there were no restrictions regarding the timing, duration, or modality of the interventions.

(c) Comparator(s)/control

The types of the studies that were included in this review were experimental (randomised controlled trials) or quasi-experimental studies (without randomised allocation). Both controlled and uncontrolled studies were considered for inclusion.

(d) Outcomes

Interventions’ primary outcome could be either asset-oriented or deficit-oriented psychological outcomes in the workplace. For this reason, included studies needed to report on at least one instrument that claimed to measure psychological well-being and/or mental well-being outcome(s). As described in our study’s protocol [50] “The effectiveness of digital interventions for psychological well-being in the workplace: a systematic review protocol” interventions’ primary outcomes could include any aspect of psychological or mental well-being of healthy adults in a work-setting, while secondary outcomes could include any other individual-level assessment.

(e) Type of studies: Included studies needed to report empirical research (i) written in English and (i) published in peer-reviewed journals or conference proceedings accompanied by full-length peer-reviewed papers. A restriction was posed to include only English-language papers due to financial and language constraints to identify and process papers in languages other than English.

#### 2.2.2. Exclusion Criteria

(f) Participants

Studies were excluded if they did not focus on working adults.

(g) Interventions

Studies were excluded if they reported interventions delivered in settings other than the participants’ workplace and if they did not report a psychological intervention.

(h) Comparator(s)/control

Studies were excluded if they reported digital interventions delivered simultaneously with other interventions without a comparison between them. Furthermore, studies were excluded if they reported case studies and cross-sectional research designs.

(i) Outcomes

Studies were also excluded if (i) they did not include relevant outcome measures, (ii) they focused primarily on the clinical treatment of mental health disorders, and/or (iii) their primary outcomes did not measure a deficit-oriented or asset-oriented psychological outcome.

(j) Type of studies

Studies were excluded if they (i) were not published in a peer-reviewed journal, (ii) were not written in the English language, (iii) reported conference abstracts (without a corresponding full-length peer-reviewed paper) or (iv) reportedunpublished research.

#### 2.2.3. Information Sources

A comprehensive literature search was conducted in July 2019 in five electronic databases (MEDLINE, Web of Science, CINAHL, PsycINFO, Cochrane Register of Controlled Trials (CENTRAL) and EMBASE) for studies published from January 1990 to July 2019.

#### 2.2.4. Search Strategy

The search strategy was pilot tested in PsychInfo and was refined and appropriately modified for each database (Supplementary Materials: Section S1 complete search strategy). Terms were searched in titles, abstracts, and keywords. Related systematic reviews were checked for the purpose of identifying other potentially eligible studies.

#### 2.2.5. Selection Process

All references were stored at the Mendeley desktop (v1.19.8 Elsevier, Amsterdam, The Netherlands) and subsequently duplicates were removed. One reviewer (MA) screened all titles and abstracts, and full texts were sought for those that remained unclear. Then, two reviewers (MA, EA) screened independently abstracts and full texts of potentially eligible studies against the studies’ eligibility criteria. A third reviewer (HB) advised on studies eligibility in a few cases that remained unclear after reviewers’ assessment.

#### 2.2.6. Data Collection Process

Data collection involved the data extracted independently by two reviewers (MA, EA) using the JBI data extraction form [51] in order to extract all relevant information from the studies (see Supplementary Material: JBI Data Extraction Form). NVivo (Version 11, QSR International Pty Ltd., Doncaster, Australia) was used to consolidate all extracted information.

#### 2.2.7. Quality Appraisal

Two reviewers independently (MA, SD) conducted the quality appraisal of the retrieved papers using the Cochrane Collaboration’s Risk of Bias [52] and the JBI Critical Appraisal Checklist for Quasi-Experimental Studies [51]. Agreement was reached through discussion and any disagreements were resolved after a second round of review.

#### 2.2.8. Synthesis

Due to the heterogeneity in the data and outcomes reported, statistical pooling of the data was not used. A narrative synthesis was performed that explored relationships between studies’ characteristics and findings as outlined by Popay et al. [53]. Moreover, evidence from RCTs was separately reviewed to assess relationships between interventions’ effectiveness and their theoretical base.

## 3. Results

### 3.1. Study Selection

**Figure 1:**
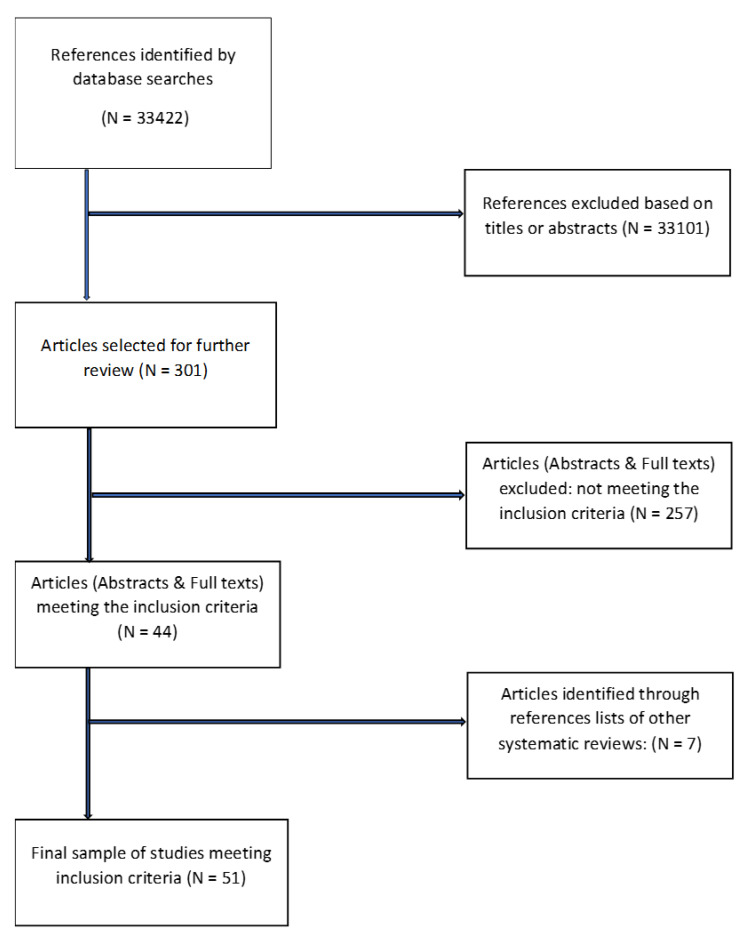
Flow diagram of studies’ selection.

The initial search identified 48,177 hits, including duplicates. In total, 33,422 articles were screened following the removal of duplicates. In total, 33,121 papers were excluded after the screening, 301 full papers were reviewed, and 51 articles met the reviews’ inclusion criteria, including 7 papers identified through references lists of other reviews (Figure 1). We excluded 257 articles for not meeting the inclusion criteria due to the following reasons: (1) not reporting relevant primary outcomes, (2) not testing a digital intervention, (3) no specific workplace settings, (4) reporting an organisational intervention, (5) simultaneous delivery of a digital intervention with other types of interventions without any comparisons, (6) not a psychological intervention, (7) not employees (e.g., university students), (8) not an RCT or quasi-experimental study, (9) paper published not in English, (10) full paper unavailable and (11) not a preliminary study.

Supplementary Materials: Table S1, study details presents the studies by their study ID number and author name, settings, intervention, population, method, measures and main findings.

### 3.2. Studies’ Characteristics

Thirty intervention studies adopted a randomised controlled trial (RCT) research design, whereas twenty-one adopted a quasi-experimental research designone. Intervention studies that used randomisation and controlled conditions involved twenty-four standard RCTs, four cluster RCTs, two pilot RCTs, and one RCT with cross-over design. Quasi-experimental intervention studies involved thirteen studies with single group pre-post designs, three pilot studies with single group pre-post designs, three non-randomised controlled trials, and two randomised trials without a control group. RCTs had an average of two hundred forty-nine participants (min = 30, max = 762) working across different sectors, including technology/information technology companies (5 RCTs), healthcare (8 RCTS), manufacturing (3 RCTs), emergency services (2 RCTs), media (1 RCT), insurance (1 RCT), education (1 RCT), civil service (1 RCT) and various companies/organisations/associations (8 RCTs). With regards to their control conditions, nineteen RCTs included control/comparison conditions. Among those, one RCT compared two different forms of a digital intervention with a control condition of not receiving an intervention, while three RCTs compared digital and in-person versions of an intervention with control conditions of not receiving an intervention or not receiving one of those. Quasi-experimental studies had an average of ninety-nine participants (min = 15, max = 379) employed as health professionals (11/21), university employees (2/21), firefighters (1/21), employees in governmental or public enterprises (3/21), white-collar employees (1/21), engineers (1/21), construction machinery employees (1/21) and naval active-duty members (1/21). Control conditions involved a waiting list (n = 2), and not receiving previously the intervention (n = 1).

### 3.3. Process of Narrative Synthesis

We first coded the studies on NVivo in terms of their characteristics, including study designs, settings and populations. Then we proceeded to code them by the type of interventions, the measures they used and the outcomes they reported. Finally, we completed our narrative synthesis by tabulating intervention outcomes per intervention type.

### 3.4. Objective 1: Description of Psychological Well-being Measures of Digital Psychological Interventions at Work

Due to the vast differences in study design and populations, the outcomes measured varied considerably. For this reason, as discussed in the study’s protocol [50], three clusters of primary outcomes and one cluster of secondary outcomes were formed:

i. Primary Outcomes
  (a) Common mental well-being outcomes at work (referred to for the rest of this review as ‘Mental health concerns’) (e.g., measures of anxiety, depression).
  (b) Work-related well-being (e.g., measures of perceived stress, stress indicators, burnout etc.).
  (c) Psychological indicators for mental well-being at work (referred to for the rest of this review as ‘Psychological wellness indicators’) (e.g., measures of psychological resources, subjective well-being etc.).
ii. Secondary Outcomes
  (d) Workplace outcomes (e.g., measures of absenteeism, job attitudes etc.)

A key difference across the three clusters of primary outcomes is the approach they adapt towards psychological well-being improvement. For those focusing on mental health concerns, improvement is equated with the reduction of negative (deficit-oriented) mental health outcomes. For those targeting work-related well-being, improvement included both deficit-oriented (e.g., distress, burnout) and asset-oriented outcomes (e.g., work-engagement). Finally, those focusing on psychological wellness indicators improvement is equated with higher levels of different psychological and psychosocial resources, subjective well-being and positive mental health.

#### 3.4.1. Description of Psychological Well-being Measures

(a) Primary Outcomes

Mental health concerns were assessed using well-established previously validated instruments measuring depression, anxiety and dysfunctional attitudes. Most frequently used instruments included different versions of the centre for epidemiologic studies depression scale: the 20-item (CES-D) scale [54,55,56,57,58] and the CESD-R-20 [59]; the Depression, Anxiety, and Stress Scale (DASS-21) [42,60], the Japanese version of Dysfunctional Attitude Scale 24 (DAS24-J) [61], the Attributional Style Questionnaire (ASQ) [62], the Hospital Anxiety and Depression Scale (HADS) [55,57,62], the Beck Anxiety Inventory (BAI) [25,59], and the Spielberger State-trait Anxiety Index (STAI) [63,64].

Assessments of work-related well-being included instruments measuring perceived stress most frequently with the 10-item PSS [58,63,65,66,67] and the Stress Questionnaire [10,56,68,69]; instruments measuring stress outcomes, psychological distress and job strain often with Job Stress Questionnaire (BJSQ) [70,71], the Symptoms of distress scale [57,59,71], and the Job Stress Questionnaire (BJSQ) [70,71]; and instruments measuring burnout, work-engagement and compassion-fatigue with the Maslach Burnout Inventory [56,69,72], the Professional Quality of Life-Revision IV (ProQOL) [73,74], and the Utrecht Work Engagement Scale [10,71,75,76,77].

Finally, assessment of psychological wellness indicators included instruments measuring general mental well-being/positive mental health such as the WHO-5 well-being scale [67,75,78,79] and the Mental Health Continuum [75,76]; as well as instruments measuring happiness and satisfaction in life most frequently using the Positive and Negative affect schedule [59,69,80], the 8-item Flourishing scale [69,78,80], and the satisfaction with Life Scale (SWLS), [80,81]. Other studies included instruments measuring mindfulness such as Freiburg Mindful-ness Inventory [63,78,82,83] and the Five Facet Mindfulness Questionnaire (FFMQ) [84,85] as well as instruments measuring psychological and psychosocial resources such as resilience with the Connor–Davidson Resilience Scale (CDRISC) [74,83,86], and various scales measuring self-efficacy [63,70,87], coping [57,87,88] and gratitude [89,90].

(b) Secondary Outcomes

Studies’ secondary outcomes involved measures of workplace outcomes. These included measures of job attitudes such as the Nurse Satisfaction Scale [57], and the Maastricht Job Satisfaction Scale for healthcare (MAS-GZ) [76], as well as measures of job performance such as the World Health Organization (WHO) Health and Work Performance Questionnaire (HPQ) [55,57,71,77], and the Work Limitations Questionnaire [57,58,59]. Other frequent assessment of workplace outcomes included measures of job attendance such as the short form World Health Organization Health and Work Performance Questionnaire [87], and self-reported sick leave days [56,77,89,91].

#### 3.4.2. Effectiveness of Digital Psychological Interventions

i. Primary Outcomes
  (a) Mental Health Concerns

Ten studies reported positive effects on mental health concerns at work. Three CBT interventions showed significant effects on depression and anxiety over time [61,62,87] and especially among those with high psychological distress at baseline [61]. Stress inoculation training [64] and relaxation training [54] showed positive effects in reducing anxiety and depression (*p* < 0.05), while two meditation training/mindfulness-based interventions [65,82] showed positive effects in reducing anxiety and depression symptoms (*p* < 0.05). Finally, one self-help intervention that delivered problem-solving therapy had small effects in anxiety [55], another that delivered problem-solving therapy had had sustainable positive effects on depressive symptom severity (*p* < 0.01) [56]; and finally another that included CBT-informed modules reported significant sustainable reduction in depression scores [60].

Seven studies reported no effects on mental health concerns at work including one CBT intervention [38], four stress-management/well-being promotions interventions [1,11,21,46], and two self-help interventions that delivered problem-solving therapy and cognitive therapy [47,48].

(b) Work-related well-being

Twenty-eight studies reported positive effects on work-related well-being measures. Two RCTs of an internet-based CBT programme had marginally significant effects on distress (*p* = 0.09) and indirect effects on work-engagement but only through changes in depression (*p*’s < 0.1) [61]. Nine studies (7 RCTs and 2 quasi-experimental studies) reported positive effects of stress-management and well-being programs on work related well-being measures including perceived stress [59,66,67,69,92,93], subjective symptoms of stress well-being [67], distress [66], job stress [47], work-related fatigue (*p* < 0.05) and psychosocial demands (mental workload, emotional labour) [54,94]. However, three RCTs [68,71,94,95] reported minimal effects on improving work-related well-being. Moreover, there were greater effects for an instructor-led workshop (F = 4.45, *p* < 0.05), while participants in both conditions were especially benefited from mini-relaxation exercises, especially after the second session (F = 8.44, *p* < 0.01) [95]. Similarly, a videoconferencing-based telepsychology intervention [68] showed greater effects for the in-person condition than the digital one. Nine studies (3 RCTs and 6 quasi-experimental studies) reported positive effects of meditation training or mindfulness-based interventions on work-related well-being measures [63,65,66,72,73,82,84,85,92]. Those included significant effects on job control (F = 5.71, *p* < 0.05), that was sustained for 2 months post-intervention [82]; a lower risk for compassion fatigue (*p* < 0.05) for nurses below the clinical cut-off point for PTSD [84]; a significant improvement for compassion fatigue and burnout (*p* < 0.05) [73]; a significant decrease in perceived stress (*p* < 0.05) [63]; small to moderate effects on stress levels post-intervention (Cohen’s d = 0.34; *p* < 0.001) that continued three months later (Cohen’s d = 0.22; *p* < 0.05) [92], and improvements in fatigue scores (*p* < 0.05) [63,65] as well as in chronic and acute fatigue scores post-intervention (*p* < 0.05) [85]. Finally, seven studies (3 RCTs, 1 pilot RCT and 3 quasi-experimental studies) reported positive effects of self-help interventions on work-related well-being measures *p* < 0.05). Those included significant effects on perceived stress [56,96] and worrying over time [56], stress levels over time [78,81,97], work engagement [96] burnout and compassion fatigue [74], and small effects on emotional exhaustion [61].

(c) Psychological wellness indicators

Nineteen studies reported positive effects on psychological indicators for mental well-being at work. Two studies, one RCT and a quasi-experimental study, reported positive effects of computerised CBT [61,87] on efficacy variables over time (*p* < 0.05) [61], as well as on self-esteem (F = 31.5; *p* < 0.05) and coping flexibility (F = 14.2; *p* < 0.001) post-intervention [87]. Four studies, three RCTs and one quasi-experimental study showed positive effects of stress-management and well-being promotion programmes [64,75,93,98] on psychological well-being (*p* < 0.01) [75,98], mental energy and active coping (*p* < 0.05) [64,91]. Seven studies, three RCTs and four quasi-experimental studies, showed significant positive effects of meditation training/mindfulness on psychological wellness indicators. Those include significant effects on psychological resources, positive emotions, and flourishing (*p* < 0.05) [63,69,73,79,82,83,85] and moderate to large effects on global mental well-being over time [82]. Finally, five self-help interventions, one RCT and four quasi-experimental studies had significant positive effects (*p* < 0.05) on positive affectivity, life satisfaction, happiness, flourishing, quality of life and self-efficacy [78,80,81,88]. However, three mobile-based resilience training interventions [74,81,99] did not have significant effects on resilience measures, while two RCTs and two quasi-experimental studies that tested three online positive psychology interventions [76,89,90] had no significant effects on any indicators of psychological wellness.

ii. Types of interventions

We grouped the interventions based on the authors’ explicit descriptions of their intervention approach and, secondarily, on key components of the interventions (i.e., mode of delivery, intervention content, and engagement with users). We identified four clusters of interventions:

(a) Psychological wellness indicators

Self-help interventions was the most frequently tested cluster of interventions (n = 18). They involved educational interventions [70,88,97,99], multicomponent interventions featuring cognitive and behavioural interventions [55,56,57,60,80,81,96], positive psychology exercises [76,78,89,90,100] and resilience training [74,101].

(b) Stress-management and workplace well-being promotion programs:

Another cluster of studies (n = 14) were ten RCTs and four quasi-experimental studies that were identified by their authors either as stress-management interventions or workplace health and mental health promotion programmes. Ten out of fourteen interventions were delivered through self-paced online sessions, five of which combined psychoeducation with training in cognitive techniques [45,59,71,95,98].

(c) Meditation training and mindfulness-based interventions:

Fourteen studies (six RCTs and eight quasi-experimental studies) reported on the effectiveness of meditation training and mindfulness-based interventions. Interventions were delivered via online platforms as educational programmes [58,63,69,72,79,83,85,86,92] and via mobile applications [65,73,82,84,102]. Mobile-based interventions delivered guided meditation practices. Other web-based interventions combined psychoeducation with training in meditation practices [69,72,79] or included online evidence-based psychological skills training sessions based on mindfulness-based stress reduction, acceptance and commitment therapy and mindfulness-based cognitive therapy [63,83,85,86,92].

(d) Cognitive Behavioural Therapy (CBT): Five studies, four RCTs and one quasi-experimental study, reported computerised, internet-based or digitally enhanced CBTs. Four interventions were delivered through self-paced computer programmes [61,62,77,91] and 4/5 included trained clinicians in some capacity.

We constructed an auxiliary evidence map (Supplementary Materials: Section S2—Evidence maps) to retain an overview of effects observed across the three clusters of primary outcomes for this review.

### 3.5. Objective 2: Relationship between Interventions’ Theoretical Base and Their Effectiveness

We evaluated RCTs’ theoretical base in order to understand the relationship between digital interventions’ effectiveness and their theoretical underpinnings. This was also in line with this review’s protocol that required the production of a separate analysis for the included RCTs. For this evaluation, we used the most relevant items from the ‘theory coding scheme’ (TSC) [103], combining them in two categories: a) Theory constructs (Are specific models/theories explicitly mentioned along with their relationship with targeted psychological constructs?), and b) Intervention components (Are intervention techniques explicitly linked to theory relevant constructs?). This analysis allowed the tabulation of interventions’ reported effects for “some effects” (only post-intervention) and “multiple effects over time” (post-intervention and follow-up) against the theoretical mechanisms described in those interventions. For those interventions that no explicit theory-base was described, the “theoretical mechanism” column was also left blank while the rest were distinguished between (i) those targeted theory-based constructs OR described theory-based intervention components, and (ii) those targeted theory-based constructs AND described theory-based intervention components (Table 1 Intervention types, theory mechanisms and effectiveness, Supplementary Materials Sections S1–S3: Full theory assessment).

The most effective cluster of interventions appeared to be theory-informed digital interventions that delivered evidence-based cognitive or behavioural training for the improvement of mental health concerns at work and other measures of work-related well-being [55,56,61,62,77,96]. Those interventions included computerised or digitally enhanced CBT targeting dysfunctional attributional styles through cognitive restructuring and self-regulation [61,62,77]; problem-solving therapy [55], and multi-component cognitive-behavioural training [56,60]. Stress-management and workplace well-being promotion programmes were often atheoretical as only 5/10 were partially explicitly based on a specific theory. Only one study [64] demonstrated a strong theoretical base as it was explicitly informed by Karasek’s job strain model [104] measuring psychosocial outcomes and showed positive effects on improving state anxiety and active coping [64]. Four in five atheoretical interventions showed some positive effects on work-related well-being measures [47,59,93,95]. Finally, three in four partially theory-informed interventions [66,67,75] showed positive effects on work-related well-being and measures of psychological wellness based on acceptance and commitment therapy [66] and broad or dynamic conceptualisations of health and positive mental health [67,75]. For example, one intervention [67] that offered a tailored choice of online positive psychology interventions, viewing them as part of the preventive role of workers’ health surveillance system that can improve subjective well-being [105], psychological well-being [106] and positive mental health [107] showed significant improvement in positive mental health but not significant differences for work-engagement, subjective well-being and mental health concerns (anxiety and depression). Mindfulness-based interventions were, in their majority, at least partially theory-based (4/6) but the impact of their theoretical assumptions was less clear. Two partially theory-informed interventions justifying meditation training on relaxation mechanisms showed no effects on stress [100,101]. In comparison, two mindfulness-based interventions that adopted Karasek’s job strain model demonstrated significant effects on different aspects of psychological well-being at work. One study [82] adopted Karasek’s model to justify measuring psychosocial outcomes, based its intervention components on the two-component model of mindfulness describing associations of mindfulness components with social support in the workplace and found significant effects sustained over time on depression, job strain, and psychological wellness measures. Another partially theory-informed mindfulness-based study that adopted Karasek’s model showed some significant effects on work-related fatigue and showed that acting with awareness fully mediated the effects of the intervention on work-related well-being [85]. Finally, a good portion of self-help interventions (6/10) was partially theory-informed [55,56,78,80,96,101]. Overall, those partially theory-informed interventions within this cluster targeted psychological constructs or justified components of their interventions based either on cognitive and behavioural techniques or approaches to happiness and positive psychology interventions [78,80,101]. Only two partially theory-informed interventions [78,80] based on positive psychology techniques showed positive effects on psychological wellness measures. Those studies adopted Lyubomirki’s theorising on how developing positive emotions, cognitions and behaviour through performing appropriately tailored activities can be associated with flourishing in the workplace [108].

### 3.6. Objective 3: Associations with Workplace Outcomes (Secondary Outcomes)

We formed a separate cluster of interventions’ secondary outcomes. Those involved effects on workplace outcomes (e.g., job attitudes, job performance, job attendance). Only two studies, two RCTs and one quasi-experimental study, reported positive intervention effects on workplace outcomes. A quasi-experimental study showed that an interactive cCBT [87] had statistically significant effects on presenteeism (post-intervention and one month follow-up). An RCT showed that a web-based CBT had marginally statistically significant effect on sick leave days during the past three months [77]. Finally, another RCT demonstrated that multi-component mental health promotion [45] that included screening, feedback and a tailored choice of online interventions had statistically significant positive effects on work functioning (*p* < 0.01).

### 3.7. Critical Appraisal

All the included studies (n = 51) were assessed for risk of bias. Agreement between reviewers was reached in two rounds following an exchange of comments on their assessment. The Cochrane handbook classification guide was followed for RCTs (n = 30) and randomised trials (n = 2). The robvis online tool was used to generate a risk-of-bias plot for studies that used randomisation [109]. (Table 2).

The greatest risks of bias were associated with small sample sizes, high attrition rates and potential contamination effects. In particular, 14/32 studies were Unclear in describing their randomisation processes, and 6/32 demonstrated insufficient or no allocation concealment. Furthermore, many studies reported high attrition levels, with 11/32 not reporting adequately any processes of managing attrition or missing values and 11/32 studies not reporting power calculations for their sample size. Others reported low power due to small samples. Overall, only a few adequately powered studies used randomisation and demonstrated low attrition bias and low risk for contamination effects [67,71,91]. Among those, only one multi-component, partially theory-informed stress-management intervention reported positive effects on work-related well-being [67]. The JBI Critical appraisal checklist for quasi-experimental studies was followed for all non-randomised intervention studies (n = 19) (Table 3).

The highest proportion of bias (6/19) involved differences between the treatment groups and differences in treatment received beyond the intervention. Studies reported comparisons between single groups at different time points that received slightly different interventions or included samples with prior exposure to or knowledge of the intervention. In addition, 9/19 studies utilised small samples (n < 40) that often faced low statistical power due to high attrition and low sample size. Only two studies showed both low risk of selection bias and low risk to exposure to other treatments, along with adequate reliability of outcome measures and appropriate statistical analysis [63,65]. Both studies reported significant effects of digital mindfulness-based interventions on fatigue scores [63,65].

## 4. Discussion

This systematic review synthesised the evidence on the effectiveness of digital psychological interventions in the workplace. Previous reviews have shown that group-based in-person psychological interventions delivered in the workplace can have small positive effects on psychological well-being and possibly improve desirable work outcomes [110,111,112]. Furthermore, group-based in-person mindfulness meditation programs may improve some physiological indices of stress among employees [63]. With most of the evidence elicited from studies reporting interventions that require in-person attendance, there is less systematic evaluation of the effects of digital psychological interventions across different facets of psychological well-being at work.

Recent meta-analyses showed that digital psychological interventions can have small positive effects on mental health, especially in reducing stress, depression symptoms, psychological distress, and improving work performance [23,42]. Moreover, app-supported CBT has been found to produce the largest effects on common mental health problems [113]. However, there is a generally fractured overview of the effectiveness of digital psychological interventions in the workplace. Reasons include a primary focus on specific intervention approaches or methods, evidence syntheses including both digitally delivered and in-person interventions, the prioritisation of deficit-based or asset-based well-being outcomes, the extensive variance in interventions’ characteristics and low-quality research designs that can limit the robustness of a synthesis’ conclusions [111,114,115]. For this reason, we conducted an integrative narrative synthesis of the evidence on effectiveness measures, including any digital psychological interventions in the workplace. Subsequently, we mapped the effects of four groups of digitally delivered psychological interventions (CBT, meditation training/mindfulness-based interventions, stress-management/well-being promotion, and self-help interventions) against three categories of outcomes: prevention or management of mental health concerns, work-related well-being outcomes, and psychological wellness indicators.

However, just five studies (3 RCTs and 2 quasi-experimental studies) [63,65,67,71,91] demonstrated a lower proportion of bias than the rest. Thus, it is important to treat any interpretations of results with caution. For example, many RCTs suffered from high attrition; however, few provided clear details on how this was mitigated. At the same time, only a small number of quasi-experimental studies offered details on completion of follow-up assessments further diminishing the quality of the evidence they provide. What is more, a significant portion of the studies targeting work-related well-being and psychological wellness indicators reported substantially more positive results than negative ones, which can be an indicator of publication bias. On the contrary, there was a relative balanced report between positive and negative results reported overall across interventions targeting mental health concerns, which is indicative of the robustness of CBT interventions.

Objective 1: Effectiveness of digitally delivered psychological interventions at work

The most frequently cited type of intervention was self-help interventions, followed by stress-management/workplace well-being promotion programmes and mindfulness-based interventions and a small cluster of online cognitive behavioural therapy. These interventions targeted three main clusters of psychological outcomes: (a) reduction or management of mental health concerns, (b) improvement of work-related well-being outcomes and (c) improvement of psychological wellness indicators. Overall interventions varied substantially in terms of duration, intervention content, and outcomes’ measures. Similarly to previous reviews, they demonstrated a high risk of bias [116] as both randomised and non-randomised studies demonstrated low power due to small sample sizes, increased risk of contamination effects, and high attrition bias.

(a) Evidence Group A: Mental Health Concerns at work

Effective digital interventions for the prevention or management of mental health concerns included primarily online CBT-based interventions with four out of five demonstrating sustainable effects in the reduction of depression systems [60,61,62,87,91] and short-term effects for the reduction of anxiety [62]. This is in accordance with evidence that app-supported CBT can produce the largest effects on common mental health problems [113]. However, there was no evidence for their effectiveness among employees with already elevated depression scores [55,57,91], which supports previous findings that online CBT in the workplace may be less suitable for the treatment of symptoms among those already suffering from depression [117,118]. The only non-CBT interventions that were effective in reducing depression symptoms were app-based mindfulness practice with the Headspace [82] and two self-help interventions delivering problem-solving therapy [56,57]. Other effective non-CBT interventions included another app-based mindfulness practice, two of six stress-management interventions that included relaxation techniques and another self-help intervention that delivered problem-solving therapy and demonstrated some positive effects in reducing anxiety [54,57,64,65]. Similarly, a meta-analysis showed that mindfulness-based interventions at work could have higher moderate effects on anxiety than depression [111]. Furthermore, a recent review on the effectiveness of mindfulness-based self-help interventions in the general population showed that they can have small effects on depression and anxiety measures post-intervention but do not seem to be retained in follow-ups [119].

(b) Evidence Group B: Work-related well-being outcomes

Psychological interventions for improving work-related well-being outcomes targeted a mixture of deficit-oriented and asset-oriented psychological outcomes. What characterised those outcomes was that they did not necessarily focus on an absence of negative mental health conditions. This was frequently the case in stress-management interventions [120] and also applied in this review to interventions targeting work-related stress and stress outcomes as part of workplace health or mental health promotion programmes, an approach frequently adopted as a prevention strategy for job stress [8,121]. For this reason, we clustered together interventions described as stress-management interventions with those that combined training in stress-management techniques with psychoeducation and other psychological skills’ training.

The most frequently utilised interventions targeted work-related well-being outcomes and were delivered either via web-based tools or mobile apps. Intervention effects included significant positive effects primarily on perceived stress [56,66,68,93,95], distress [45,59,66], subjective symptoms of stress [67], work-related fatigue [45], as well as in psychosocial demands (i.e., emotional labour, mental workload) [54,94] and work-engagement [75]. Psychoeducation alone showed only some effects in the management of emotional labour and mental workload. It had, though, no effects on any other work-related well-being outcomes, which is in line with previous reviews of the literature showing educational interventions to be the least effective for employees’ psychological health [122,123,124]. Relaxation training appeared to be an essential characteristic of effective interventions for stress-management and well-being promotion programmes [54,67,68,93,95], while tailored brief interventions that were part of e-mental health programmes showed marginal or no effects on perceived stress [45,59]. Stress inoculation training and relaxation techniques [54,64] were the only stress-management interventions that showed positive effects in reducing mental health concerns. Previous reviews have also confirmed the existence of strong evidence for the effectiveness of physical relaxation and mindfulness practice for reducing occupational stress [13,125]. However, a systematic review of brief mental health and well-being interventions found no evidence for their effectiveness [116]. What is more, mental health promotion programmes and web-based and app-based stress-management interventions demonstrated significant effects on psychological wellness (i.e., positive mental health, Ryff’s Psychological well-being scale) and psychological resources (i.e., coping skill, mental energy, concentration ability and active coping) [64,67,69,93,98] but there were no significant effects on resilience [27,54]. Finally, there was evidence that in-person stress-management interventions may be more effective if not equal to digitally delivered ones [54,68,95]. There is generally less clarity on the overall effectiveness or non-inferiority of digital interventions for improving work-related well-being compared to face-to-face interventions. Nigaru et al. [126] showed that virtually delivered CBT and non-CBT might have a greater effect in reducing depression symptoms than their in-person counterparts [126], while Carolan et al.’s [23] meta-analysis of web-based psychological interventions showed that their effect on psychological well-being is comparable to non-digital workplace interventions. Finally, Vanhove et al.’s [11] review showed that face-to-face and group-based resilience-building interventions in the workplace might be more effective in improving work-related well-being outcomes than computer-based interventions.

Meditation training and mindfulness-based interventions mainly had significant effects on fatigue-related measures, a finding that has also been previously confirmed among other populations such as cancer survivors [127,128]. Mindfulness-based interventions demonstrated greater variety in delivering methods ranging from self-guided app-based training practice [65,73,82] to instructor-led courses [58,72]. Evidence from quasi-experimental studies and a few RCTs [82,85] suggests that mindfulness-based interventions and guided meditation training or mindfulness practices [85,92] often through mobile applications (i.e., Headspace, wearable neurofeedback system managed via smartphone) [65,73,82] and web-based tools [85,92] may have a significant effect on work-related fatigue. In particular, our synthesis showed significant effects on fatigue scores, compassion fatigue, chronic fatigue and acute fatigue [65,73,84,85] as well as burnout [72,84], job strain [82], and over-commitment [63] especially for those below the threshold for PTSD [84]. Meditation training and mindfulness-based interventions with the strongest evidence-based (i.e., MBSR, third-wave CBT) had the strongest effect on psychological wellness indicators (i.e., resilience, optimism, coping, mindfulness, subjective well-being, acting with awareness, daily positive emotions, gratitude, flourishing, self-compassion) [63,69,73,79,82,83,85], while half of the interventions that had significant effects on such outcome measures were also among those that reported positive effects on work-related well-being measures (i.e., Headspace app, MBSR, yoga therapy). Such findings are in accordance with growing evidence supporting the premise for mindfulness interventions in the workplace for employees’ well-being [129]. However, a recent meta-analysis showed that non-digitally delivered mindfulness-based self-help interventions have greater effects on psychological well-being measures at work than digital interventions [119]. A recent review of mindfulness-based interventions showed that they could be effective, especially among healthcare professionals, due to the high risk of burnout and the associations between mindfulness, compassion, and self-compassion [130]. In our synthesis, four out of five quasi-experimental studies reporting effective meditation training or mindfulness-based interventions targeting work-related well-being outcomes focused on healthcare professionals [63,72,73,84]. Only one quasi-experimental study, though, assessed their effects on self-compassion [79], whose associations with compassion fatigue and burnout have been increasingly the focus of discussions about healthcare professionals’ work-related well-being [130,131,132].

Digital self-help interventions for improving work-related well-being included delivering psychological skills training primarily via web-based tools. The evidence on web-based self-help interventions suggests that guided [56] and unguided [55,78,96] self-help courses delivering evidence-based cognitive-behavioural training (problem-solving, cognitive therapy, positive psychological states’ development) can improve perceived stress and emotional stress over time [78]. Only two self-help interventions were delivered via mobile applications [74,81]. They were tested using quasi-experimental research designs, and they showed that mental wellness training based on acceptance and commitment therapy [81] and resilience training [74] might improve ratings of stress [81], burnout and compassion fatigue. Still, there were no effects on compassion satisfaction [74]. Similarly to stress-management interventions, psychoeducation alone [70] showed no effect on work-related well-being measures. There was also no evidence for the effects of self-directed micro-tasks [100] on work-related well-being. Digitally delivered CBT programs only showed marginally significant effects on distress [61] and only marginally significant indirect effects on work-engagement through changes in depression [77]. However, work-related well-being measures were viewed as secondary intervention outcomes for both interventions. A meta-analysis [133] points out that CBT-based interventions may be more suitable for addressing stress manifestations than others, while intervention settings can be a significant moderator of observed intervention efficacy with group-based interventions to demonstrate stronger effects on exhaustion.

(c) Evidence Group C: Psychological wellness indicators

Interventions that focused on the improvement of psychological wellness indicators targeted asset-oriented outcomes. This cluster of interventions was also the one with the lower quality of evidence, especially for the effectiveness of self-help interventions.

Digital mindfulness-based and self-help interventions frequently targeted psychological wellness indicators. Studies described a variety of online- and app-based interventions and training courses that demonstrated positive effects on positive affect, life satisfaction, happiness and flourishing, pandemic self-efficacy and perceived workplace resilience. A review of digital self-help interventions in the overall population showed that they could positively affect mental well-being (e.g., mood enhancement) but only if they have adequate uptake and adherence [134]. However, Van Agteren et al.’s [135] meta-analysis showed substantial differences in the quality of the evidence of psychological interventions for mental well-being in the general population. For example, they found high-quality evidence for small to moderate effects for mindfulness-based interventions, low-quality evidence of limited effects for multi-theoretical interventions, and superiority of group-based interventions over individual-based and technology-based interventions. Our synthesis also signals that other intervention-relevant or population-relevant characteristics need to be addressed to explain differences in interventions’ effectiveness fully. One quasi-experimental study combining psychoeducation with evidence-based psychological strategies, including acceptance and commitment therapy, showed non-significant improvement in resilience and psychological flexibility [86]. What is more, two quasi-experimental studies [69,79] showed positive effects of online hourly training modules on a variety of psychological wellness indicators (i.e., gratitude, self-compassion, flourishing, positive and negative affect). Moreover, two RCTs showed no effects of real-time virtual classes and an online training programme on mindfulness [58,102]. What is more, there was no evidence of interventions’ effectiveness either using psychoeducation solely or combining it with positive psychology exercises for improving dispositional resilience or other psychological wellness indicators (i.e., happiness, gratitude, job-related affective well-being) irrespectively to their theory-base. Finally, two studies reported that secondary outcomes of interactive CBT programmes involved positive effects on efficacy variables, coping flexibility and self-esteem [61,87].

Objective 2: Relationships between interventions’ theory-base and their effectiveness

We evaluated the theory-base of all included RCTs using an adaptation of the ‘theory coding scheme’ (TSC) categories [103], which has also been used to evaluate the theoretical basis of psychological and occupational health interventions [136,137]. Our results showed that although many of those interventions were merely partially theory informed, they were still more effective than those that were not theory-informed. However, the actual relationship between interventions’ theory base and effectiveness differed across different outcome measures. Previous research has shown that theory-informed interventions, often based on self-determination theory, can have positive effects on mental health self-management [138,139]. However, much less is clear about the effects of theory-informed psychological intervention on individuals’ psychological health self-management in the workplace. The most effective interventions for reducing depressive symptoms and anxiety were both theory-informed and evidence-based interventions. Those involved online CBT targeting dysfunctional attributional styles through cognitive restructuring and self-regulation [61,62,77]; problem-solving therapy [55,56] and multi-component cognitive-behavioural training [60]. The only other RCTs that showed effects on such mental health concerns involved training in mindfulness practices using the Headspace mobile application [82] and mobile stress inoculation training [64]. Those were both informed by Karasek’s job strain model for targeting specific psychological outcomes.

As far as work-related well-being is concerned, there was a weaker association between intervention effectiveness and the strength of their theory base as many atheoretical stress-management interventions showed significant positive effects primarily on perceived stress [47,59,68,93,95]. At the same time, none of the effective partially theory-informed interventions [66,67] fully described intervention techniques explicitly linked to theory-relevant constructs (e.g., teaching ACT principles without relating them to specific intervention components) [66]. Furthermore, two out of three mindfulness-based interventions that reported effects on work-related well-being measures was at least partially theory informed. This finding suggests that the theory-base of intervention mechanisms [82,85] and targeting theory-relevant psychological constructs [82] may be associated with the effectiveness of mindfulness interventions. On the other hand, effective self-help interventions were often partially theory-informed. They frequently incorporated problem-solving therapy, self-regulation, and cognitive therapy and had significant effects on perceived stress [56,96] and emotional exhaustion [55]. On the contrary, all three RCTs (two meditation training interventions and one self-help intervention) [58,100,102] showing no effects at all for work-related well-being were also among those that were judged as being not theory-informed.

Contrary to studies targeting work-related well-being outcomes, the majority of RCTs reporting positive effects on psychological wellness measures were at least partially theory-informed [64,67,75]. Theoretical mechanisms of effective interventions demonstrating multiple effects over time involved Lyubomirki’s theorising on positive emotions’ development and goal setting and planning theory (i.e., intentional activities, to cultivate positive feelings, setting and pursuing goals) [78,80] that explained the intervention mechanisms described in those studies although at times with less direct links with specific outcome measures (i.e., flourishing). Similarly to studies targeting mental health concerns, some of the most effective interventions with the strongest theory-base were those that used Karasek’s job strain model [64,82]. They adopted that model to explain the targeted effects of stress inoculation training or justify targeting psychosocial outcomes (i.e., social support) and provide an explicit description of how intervention components (i.e., relaxation techniques, regulation of attention) are linked with the theoretical construct of job strain [64,82]. Finally, some theoretical approaches that were associated with effective interventions although, with less clear links to intervention outcomes were mindfulness mechanisms of change or the facets of mindfulness [67,85] and dynamic concepts of health and wellness for positive mental health or psychological well-being [69,75,80].

Objective 3: Associations with workplace outcomes

The included studies in our review suggest minimal associations between their intervention effects and workplace outcomes. Those involved effects of online CBT on presenteeism and marginally on the number of sick days. However, they were assessed as secondary intervention outcomes [77,87]. Finally, there were some effects of an online well-being promotion programme on work-functioning that was a line of enquiry generated from previous trial arms focusing specifically on work performance [45]. These findings are in accordance with recent findings that workplace interventions have a weaker impact on workplace outcomes than mental health [114].

## 5. Limitations

There are a number of limitations that also need to be acknowledged. First of all, a significant portion of the included studies demonstrated low quality. Furthermore, we acknowledge that the inclusion of additional reviewers at the abstracts/full-papers screening and the quality appraisal stage but not at the titles/abstract screening stage could have influenced the overall quality of the screening process. Due to the large heterogeneity in intervention characteristics and research designs of studies evaluating digitally delivered psychological interventions in the workplace, we included only randomised controlled trials and quasi-experimental studies in the review, thus evidence from qualitative or mixed methods studies was excluded. Our synthesis showed that many digital mindfulness-based interventions, which have gained popularity in recent years, have not been evaluated using RCT designs. This means that conclusions about their effectiveness may be more problematic than the evidence from digital interventions delivering cognitive behavioural therapy or cognitive-behavioural skills training. However, excluding them would significantly reduce the scope of emerging evidence in the field. Furthermore, the evidence on digital third-wave CBT interventions still lags behind comparatively to other digitally delivered psychological interventions and for this reason we refrained from clustering together studies that included such components. Similarly, emergent digital interventions in the field that have been examined via other methods (e.g., qualitative or mixed methods research designs) were essentially excluded by this review. Furthermore, the review was limited to articles published in English. Thus, relevant literature published in another language may have been missed. Moreover, the searches for this review were completed in July 2019; thus, it only includes studies published before the COVID-19 pandemic. A future update of this review could examine changes in our knowledge on the effectiveness of digitally delivered psychological interventions in the workplace post-pandemic. Finally, our review explicitly focused on interventions delivered in the workplace; thus, studies that followed open community recruitment processes were not included. Previous research has shown that such recruitment strategies may heighten the effectiveness of occupational e-mental health interventions compared to workplace recruitment [140], which further highlights that there may be ‘unknown’ mediators that may count for interventions’ observed effectiveness or its absence.

## 6. Conclusions

A key finding of this review is that evidence-based interventions that aim to improve psychological well-being in the workplace can be significantly benefited by adopting a clear theoretical framework that informs both the content of the intervention and its targeted outcomes. What is more, more research needs to be directed towards comparing directly digital interventions with equivalent in-person interventions. This also highlights the importance of adopting strategies to capture small differentiations between interventions, such as recording participant preferences before their random assignment to one or another condition and incorporating those in subsequent modelling to address their effect as a confounder or mediator, on a study’s targeted outcomes [141]. Some key recommendations, though, based on our review, are the following:

- Digitally delivered CBT, problem-solving, relaxation techniques, stress inoculation training and meditation practice using the Headspace mobile application can inform well-being programmes to prevent the development of mental health concerns at work.
- Training in relaxation techniques is an essential element for effective stress-management interventions at work, and interventions targeting occupational stress may benefit in-person delivery methods.
- Psychoeducation alone is the least effective intervention approach for psychological well-being promotion in the workplace
- Theory-informed digital interventions are associated with greater effectiveness

## Figures and Tables

**Table 1:** Intervention types, theory mechanisms and effectiveness.

	No Effects	Some Effects	Theory Mechanisms	Multiple Effects Over Time (T3 Follow-Up)	Theory Mechanisms
No theory-base	5 RCTs: (85) cCBT (95) self-help (96) self-help (91) self-help	6 RCTs: (86) Mindfulness-based (66) mindfulness-based (64) Stress management (47) stress management (65) stress management (72) stress management (37) stress management/health promotion	-	1 pilot RCT: (63) self-help: multi-component intervention	
Theory-based constructs and theory-based intervention components	0 RCTs	2 RCTs (54) cCBT (57) Stress management	(45) attributional style and cognitive and behavioural skills to improve it and reduce depression symptoms (34) Karasek’s stress model and stress inoculation training (relaxation effect)	2 RCTs (55) cCBT (59) Mindfulness-based	(55) cognitive restructuring and dysfunctional attitudes (59) Karasek’s stress model-mindfulness components and social support
Theory-based constructs or theory-based interventions	4 RCT (101) Minduflness-based (100) Minduflness-based (71) Stress management (social learning theory) (97) self-help: positive psychology interventions	6 RCTs (83) Mental health promotion (62) self-help:problem-solving (102) cCBT (78) self-help: self-regulation (68) Stress management (61) self-help: problem-solving (76) Meditation and mindfulness-based	(83) psychological well-being measures (positive mental health, subjective well-being measures), and positive psychology interventions choice (autonomy-self-determination theory) (62) Problem-solving therapy (102) cCBT (cognitive restructuring) (78) self-regulation training (68) ACT mechanisms (61) Problem-solving therapy (76) Mindfulness mechanisms of change, affective rumination and problem solving pondering and measures	3 RCTs (88) Self-help: positive psychology (80) Self-help: positive psychology (69) Stress management	(88) psychological well-being measure (flourishing measure) and goal setting-happiness (80) Lubomirsky’s happiness approach and positive psychology interventions for cultivating positive feelings, positive behaviours, or positive cognitions (69) mechanisms actions of mindfulness, relaxation response and positive mental health

**Table 2:** RCTs quality appraisal *.

Study ID	Risk of Bias						
	D1	D2	D3	D4	D5	D6	D7
1	x	x	x	-	+	+	x
5	-	+	x	-	-	+	x
8	x	x	x	-	-	+	-
9	-	+	x	-	-	+	-
10	x	-	x	-	+	+	-
11	x	-	-	-	-	+	x
12	+	+	-	-	+	+	x
14	+	+	-	-	-	+	-
15	x	-	x	-	+	+	-
16	x	+	x	-	-	+	-
18	x	+	x	-	x	x	+
20	x	+	+	-	x	+	x
22	x	+	x	-	-	+	-
23	+	-	x	-	-	+	+
26	x	+	-	-	x	+	-
28	x	-	-	-	+	-	-
32	x	x	x	-	+	+	-
33	x	+	x	-	+	+	x
34	x	x	x	-	-	-	x
37	x	-	x	-	+		-
38	x	+	+	-	+		+
39	x	x	x	-	+		x
40	x	+	x	-	+		-
42	x	-	-	-	-	-	x
43	x	-	x	-	+		x
44	x	-	x		+		x
45	x	+	x	-	-	-	-
47	x	+	x	-	+		-
48	x	+	x	-	+		+
49	x	+	x	-	+		-
50	x	+	-	-	+		+
51	x	x	x	-	+		-
	D1: Random sequence allocation D2: Allocation concealment D3: Blinding of participants and personnel D4: Blinding of outcome assessment D5: Incomplete outcome data D6: Selective reporting D7: Other bias			Judgement			
				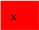 High			
				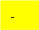 Unclear			
				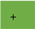 Low			

* See column 1—Table S1 in Supplementary Materials for study numbers shown within the quality appraisal table.

**Table 3:** near here. Quality appraisal of quasi-experimental studies *.

	Q1	Q2	Q3	Q4	Q5	Q6	Q7	Q8	Q9
2	+	+	-	x	+	x	n/a	-	-
3	+	+	n/a	x	+	+	n/a	+	-
4	+	+	-	x	+	n/a	n/a	+	-
6	+	+	-	+	+	-	+	+	x
7	+	+	x	x	+	x	n/a	-	-
13	-	-	x	x	-	-	n/a	-	-
17	+	+	+	x	+	x	n/a	+	-
19	-	-	x	x	+	n/a	n/a	-	-
21	+	+	+	x	+	x	n/a	-	+
24	+	+	+	x	+	+	n/a	+	+
25	+	+	+	x	+	+	n/a	+	x
27	+	+	x	x	+	x	n/a	-	+
29	+	+	+	x	+	-	n/a	+	+
30	+	+	+	x	+	x	n/a	-	-
31	+	-	+	x	x	x	-	-	x
35	+	x	x	x	+	x	n/a	+	+
36	+	+	+	x	+	x	n/a	x	-
41	+	+	x	+	+	x	+	+	-
46	+	+	+	x	+	+	n/a	+	-
	Q1:” “Cause” and “effect” Q2: Similar comparisons Q3: Receiving same treatment Q4: Control group Q5: Pre-post Q6: Follow up completion Q7: Outcomes included Q8: Reliability Q9: Appropriate Statistical analysis					Judgement			
						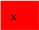 High			
						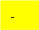 Unclear			
						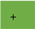 Low			
						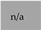 Not applicable			

* See column 1—Table S1 in Supplementary Materials for study numbers shown within the quality appraisal table.

## Data Availability

The study did not report any primary data.

## Supplementary Materials

The following supporting information can be downloaded at: https://www.mdpi.com/article/10.3390/ejihpe12100102/s1, Table S1: Studies details. Section S1: Search Strategy. Section S2: Evidence Map. Section S3: Full theory-base assessment. Section S4: JBI assessment-both reviewers.

## References

[1] Deloitte (2020). Mental Health and Employers Refreshing the Case for Investment. Deloitte. https://www2.deloitte.com/content/dam/Deloitte/uk/Documents/consultancy/deloitte-uk-mental-health-and-employers.pdf.

[2] HSE (2019). Health and Safety at Work. Summary Statistics for Great Britain 2019. HSE. https://www.hse.gov.uk/statistics/overall/hssh1819.pdf.

[3] HSE (2020). Work-Related Stress, Anxiety or Depression Statistics in Great Britain, 2020. HSE. https://www.hse.gov.uk/statistics/causdis/stress.pdf.

[4] NHS (2017). NHS Staff Survey. NHS. https://www.nhsstaffsurveys.com/static/aad7e16d5d1e244005c435d1ee12cf13/ST17_Making-sense-of-your-Staff-Survey-data.pdf.

[5] NHS (2019). NHS Staff Survey. NHS. https://www.nhsstaffsurveys.com/static/85f2245a436dbd3a1c2748fc57c4459d/Technical-document_V2.pdf/.

[6] NHS (2020). NHS Staff Survey. NHS. https://www.nhsstaffsurveys.com/static/46d36a39605945922b852508ac2ea602/NHS-Staff-Survey-2020-Technical-document-V2.pdf.

[7] Corbière M., Shen J., Rouleau M., Dewa C.S. (2009). A systematic review of preventive interventions regarding mental health issues in organizations. Work.

[8] Tetrick L.E., Winslow C.J. (2015). Workplace stress management interventions and health promotion. Annu. Rev. Organ. Psychol. Organ Behav..

[9] Richardson K.M., Rothstein H.R. (2008). Effects of occupational stress management intervention programs: A meta-analysis. J. Occup. Health Psychol..

[10] Bhui K.S., Dinos S., Stansfeld S.A., White P.D. (2012). A synthesis of the evidence for managing stress at work: A review of the reviews reporting on anxiety, depression, and absenteeism. J. Environ. Public Health.

[11] Vanhove A.J., Herian M.N., Perez A.L., Harms P.D., Lester P.B. (2016). Can resilience be developed at work? A meta-analytic review of resilience-building programme effectiveness. J. Occup. Organ. Psychol..

[12] LaMontagne A.D., Martin A., Page K.M., Reavley N.J., Noblet A.J., Milner A.J., Keegel T., Smith P.M. (2014). Workplace mental health: Developing an integrated intervention approach. BMC Psychiatry.

[13] Holman D., Johnson S., O’Connor E., Diener E., Oishi S., Tay L. (2018). Stress management interventions: Improving subjective psychological well-being in the workplace. Handbook of Well-Being.

[14] Murphy L.R., Sauter S.L. (2003). The USA perspective: Current issues and trends in the management of work stress. Aust. Psychol..

[15] Parks K.M., Steelman L.A. (2008). Organisational wellness programs: A meta-analysis. J. Occup. Health Psychol..

[16] Richardson K.M. (2017). Managing employee stress and wellness in the new millennium. J. Occup. Health Psychol..

[17] Memish K., Martin A., Bartlett L., Dawkins S., Sanderson K. (2017). Workplace mental health: An international review of guidelines. Prev. Med..

[18] Nexø M.A., Kristensen J.V., Grønvad M.T., Kristiansen J., Poulsen O.M. (2018). Content and quality of workplace guidelines developed to prevent mental health problems: Results from a systematic review. Scand. J. Work Environ. Health.

[19] Ryff C.D., Singer B.H., Dienberg Love G. (2004). Positive health: Connecting well–Being with biology. Philos. Trans. R Soc. Lond. B Biol. Sci..

[20] Wright E.M. (2018). Evaluation of a web-based holistic stress reduction pilot program among nurse-midwives. J. Holist Nurs..

[21] Warr P.B., Kahneman D., Diener E., Schwarz N. (1999). Well-being and the workplace. Well-Being: The Foundations of Hedonic Psychology.

[22] Ilies R., Aw S.S., Pluut H. (2015). Intraindividual models of employee well-being: What have we learned and where do we go from here?. Eur. J. Work Organ. Psychol..

[23] Carolan S., Harris P.R., Cavanagh K. (2017). Improving employee well-being and effectiveness: Systematic review and meta-analysis of web-based psychological interventions delivered in the workplace. J. Med. Internet Res..

[24] Robertson I.T., Cooper C.L. (2010). Full engagement: The integration of employee engagement and psychological well-being. LODJ.

[25] Böckerman P., Bryson A., Ilmakunnas P. (2012). Does high involvement management improve worker wellbeing?. J. Econ. Behav. Organ..

[26] Youssef C.M., Luthans F. (2007). Positive organisational behavior in the workplace: The impact of hope, optimism, and resiliency. J. Manag..

[27] Knight C., Patterson M., Dawson J. (2017). Building work engagement: A systematic review and meta-analysis investigating the effectiveness of work engagement interventions. J. Organ. Behav..

[28] Di Fabio A. (2017). Positive healthy organizations: Promoting well-being, meaningfulness, and sustainability in organizations. Front Psychol..

[29] Cartwright S., Cooper C.L. (2014). Towards organisational health: Stress, positive organisational behavior, and employee well-being. Bridging Occupational, Organizational and Public Health.

[30] Huang J., Wang Y., You X. (2016). The job demands-resources model and job burnout: The mediating role of personal resources. Curr. Psychol..

[31] Bakker A.B., Demerouti E. (2018). Multiple levels in job demands-resources theory: Implications for employee well-being and performance. Handbook of Well-Being.

[32] Halbesleben J.R., Neveu J.P., Paustian-Underdahl S.C., Westman M. (2014). Getting to the “COR” understanding the role of resources in conservation of resources theory. J. Manag..

[33] Day A., Nielsen K., Fraccaroli F., Sverke M., Day A., Nielse K., Chmiel N., Fraccaroli F., Sverke M. (2017). What does our organization do to help our well-being? Creating healthy workplaces and workers. An Introduction to Work and Organisational Psychology: An International Perspective.

[34] Nielsen K., Nielsen M.B., Ogbonnaya C., Känsälä M., Saari E., Isaksson K. (2017). Workplace resources to improve both employee well-being and performance: A systematic review and meta-analysis. Work Stress.

[35] Nielsen K., Yarker J., Munir F., Bültmann U. (2018). IGLOO: An integrated framework for sustainable return to work in workers with common mental disorders. Work Stress.

[36] Schaufeli W.B. (2017). Applying the job demands-resources model. Organ. Dyn..

[37] Yunus W.M., Musiat P., Brown J.S. (2018). Systematic review of universal and targeted workplace interventions for depression. Occup. Environ. Med..

[38] Delany K. (2021). What challenges will organisations face transitioning for the first time to the new normal of remote working?. Hum. Resour. Dev. Int..

[39] Naslund J.A., Bondre A., Torous J., Aschbrenner K.A. (2020). Social media and mental health: Benefits, risks, and opportunities for research and practice. J. Technol. Behav. Sci..

[40] Le L.K.-D., Esturas A.C., Mihalopoulos C., Chiotelis O., Bucholc J., Chatterton M.L., Engel L. (2021). Cost-effectiveness evidence of mental health prevention and promotion interventions: A systematic review of economic evaluations. PLoS Med..

[41] Taylor W.C., Williams J.R., Harris L.E., Shegog R. (2021). Computer prompt software to reduce sedentary behavior and promote physical activity among desk-based workers: A systematic review. Hum. Factors.

[42] Stratton E., Lampit A., Choi I., Calvo R.A., Harvey S.B., Glozier N. (2017). Effectiveness of eHealth interventions for reducing mental health conditions in employees: A systematic review and meta-analysis. PLoS ONE.

[43] Michie S., Yardley L., West R., Patrick K., Greaves F. (2017). Developing and evaluating digital interventions to promote behavior change in health and health care: Recommendations resulting from an international workshop. J. Med. Internet Res..

[44] Hekler E.B., Michie S., Pavel M., Rivera D., Collins L., Jimison H.B., Garnett C., Parral S., Spruijt-Metz D. (2016). Advancing models and theories for digital behavior change interventions. Am. J. Prev. Med..

[45] Ketelaar S.M., Nieuwenhuijsen K., Bolier L., Smeets O., Sluiter J.K. (2014). Improving work functioning and mental health of health care employees using an e-mental health approach to workers’ health surveillance: Pretest–posttest study. Saf. Health Work.

[46] Spadaro K.C., Hunker D.F. (2016). Exploring the effects of an online asynchronous mindfulness meditation intervention with nursing students on stress, mood, and cognition: A descriptive study. Nurs. Educ. Today.

[47] Hersch R.K., Cook R.F., Deitz D.K., Kaplan S., Hughes D., Friesen M.A., Vezina M. (2016). Reducing nurses’ stress: A randomized controlled trial of a web-based stress management program for nurses. Appl. Nurs. Res..

[48] Ryan C., Bergin M., Chalder T., Wells J.S. (2017). Web-based interventions for the management of stress in the workplace: Focus, form, and efficacy. J. Occup. Health.

[49] Kersemaekers W., Rupprecht S., Wittmann M., Tamdjidi C., Falke P., Donders R., Speckens A., Kohls N. (2018). A workplace mindfulness intervention may be associated with improved psychological well-being and productivity. A preliminary field study in a company setting. Front. Psychol..

[50] Armaou M., Konstantinidis S., Blake H. (2020). The effectiveness of digital interventions for psychological well-being in the workplace: A systematic review protocol. Int. J. Environ. Res. Public Health.

[51] Joana Briggs Institute (JBI) (2017). The Joanna Briggs Institute Critical Appraisal Tools for Use in JBI Systematic Reviews-Checklist for Quasi-Experimenal Studies (non-Randomised Experimental Studies). JBI. https://jbi.global/sites/default/files/2019-05/JBI_Quasi-Experimental_Appraisal_Tool2017_0.pdf.

[52] Higgins J.P.T., Altman D.G., Gøtzsche P.C., Jüni P., Moher D., Oxman A.D., Savović J., Schulz K.F., Weeks L., Sterne J.A.C. (2011). The Cochrane Collaboration’s tool for assessing risk of bias in randomised trials. BMJ.

[53] Popay J., Roberts H., Sowden A., Petticrew M., Arai L., Rodgers M., Britten N., Roen K., Duffy S. (2006). Guidance on the Conduct of Narrative Synthesis in Systematic Reviews.

[54] Baek J.H., Kim J.H., Oh S., Kim J.Y., Baik S. (2018). Smart stress care: Usability, feasibility and preliminary efficacy of fully automated stress management application for employees. Psychiatry Investig..

[55] Geraedts A.S., Kleiboer A.M., Wiezer N.M., van Mechelen W., Cuijpers P. (2014). Short-term effects of a web-based guided self-help intervention for employees with depressive symptoms: Randomized controlled trial. J. Med. Int. Res..

[56] Ebert D.D., Lehr D., Boß L., Riper H., Cuijpers P., Andersson G., Thiart H., Heber E., Berking M. (2014). Efficacy of an internet-based problem-solving training for teachers: Results of a randomized controlled trial. Scand. J. Work Environ. Health.

[57] Geraedts A.S., Kleiboer A.M., Twisk J., Wiezer N.M., van Mechelen W., Cuijpers P. (2014). Long-term results of a web-based guided self-help intervention for employees with depressive symptoms: Randomized controlled trial. J. Med. Internet Res..

[58] Wolever R.Q., Bobinet K.J., McCabe K., Mackenzie E.R., Fekete E., Kusnick C.A., Baime M. (2012). Effective and viable mind-body stress reduction in the workplace: A randomized controlled trial. J. Occup. Health Psychol..

[59] Billings D.W., Cook R.F., Hendrickson A., Dove D.C. (2008). A web-based approach to managing stress and mood disorders in the workforce. J. Occup. Environ. Med..

[60] Hirsch A., Luellen J., Holder J.M., Steinberg G., Dubiel T., Blazejowskyj A., Schladweiler K. (2017). Managing depressive symptoms in the workplace using a web-based self-care tool: A pilot randomized controlled trial. JMIR Res. Protoc..

[61] Imamura K., Kawakami N., Furukawa T.A., Matsuyama Y., Shimazu A., Umanodan R., Kawakami S., Kasai K. (2014). Effects of an internet-based cognitive behavioral therapy (iCBT) program in Manga format on improving subthreshold depressive symptoms among healthy workers: A randomized controlled trial. PLoS ONE.

[62] Grime P.R. (2004). Computerized cognitive behavioural therapy at work: A randomized controlled trial in employees with recent stress-related absenteeism. Occup. Med..

[63] Heckenberg R.A., Hale M.W., Kent S., Wright B.J. (2019). An online mindfulness-based program is effective in improving affect, over-commitment, optimism and mucosal immunity. Physiol. Behav..

[64] Villani D., Grassi A., Cognetta C., Toniolo D., Cipresso P., Riva G. (2013). Self-help stress management training through mobile phones: An experience with oncology nurses. Psychol. Serv..

[65] Crivelli D., Fronda G., Venturella I., Balconi M. (2019). Stress and neurocognitive efficiency in managerial contexts: A study on technology-mediated mindfulness practice. Int. J. Workplace Health Manag..

[66] Ly K.H., Asplund K., Andersson G. (2014). Stress management for middle managers via an acceptance and commitment-based smartphone application: A randomized controlled trial. Internet Interv..

[67] Coelhoso C.C., Tobo P.R., Lacerda S.S., Lima A.H., Barrichello C.R.C., Amaro Jr E., Kozasa E.H. (2019). A new mental health mobile app for well-being and stress reduction in working women: Randomized controlled trial. J. Med. Internet Res..

[68] Kim J.I., Yun J.-Y., Park H., Park S.-Y., Ahn Y., Lee H., Kim T.-K., Yoon S., Lee Y.-J., Oh S. (2018). A mobile videoconference-based intervention on stress reduction and resilience enhancement in employees: Randomized controlled trial. J. Med. Int. Res..

[69] Kemper K.J., Rao N. (2017). Brief online focused attention meditation training: Immediate impact. J. Evid Complement. Altern Med..

[70] Shimazu A., Kawakami N., Irimajiri H., Sakamoto M., Amano S. (2005). Effects of web-based psychoeducation on self-efficacy, problem solving behavior, stress responses and job satisfaction among workers: A controlled clinical trial. J. Occup. Health.

[71] Umanodan R., Shimazu A., Minami M., Kawakami N. (2014). Effects of computer-based stress management training on psychological well-being and work performance in Japanese employees: A cluster randomized controlled trial. Ind. Health.

[72] Leary S., Weingart K., Topp R., Bormann J. (2018). The effect of mantram repetition on burnout and stress among VA staff. Workplace Health Saf..

[73] Heeter C., Lehto R., Allbritton M., Day T., Wiseman M. (2017). Effects of a technology-assisted meditation program on healthcare providers’ interoceptive awareness, compassion fatigue, and burnout. J. Hosp. Palliat. Nurs..

[74] Wood A.E., Prins A., Bush N.E., Hsia J.F., Bourn L.E., Earley M.D., Walser R.D., Ruzek J. (2017). Reduction of burnout in mental health care providers using the provider resilience mobile application. Community Ment. Health J..

[75] Bolier L., Ketelaar S.M., Nieuwenhuijsen K., Smeets O., Gärtner F.R., Sluiter J.K. (2014). Workplace mental health promotion online to enhance well-being of nurses and allied health professionals: A cluster-randomized controlled trial. Internet Interv..

[76] Kloos N., Drossaert C.H., Bohlmeijer E.T., Westerhof G.J. (2019). Online positive psychology intervention for nursing home staff: A cluster-randomized controlled feasibility trial of effectiveness and acceptability. Int. J. Nurs. Stud..

[77] Imamura K., Kawakami N., Furukawa T.A., Matsuyama Y., Shimazu A., Umanodan R., Kawakami S., Kasai K. (2015). Effects of an internet-based cognitive behavioral therapy intervention on improving work engagement and other work-related outcomes: An analysis of secondary outcomes of a randomized controlled trial. J. Occup. Environ. Med..

[78] Feicht T., Wittmann M., Jose G., Mock A., Von Hirschhausen E., Esch T. (2013). Evaluation of a seven-week web-based happiness training to improve psychological well-being, reduce stress, and enhance mindfulness and flourishing: A randomized controlled occupational health study. Evid Based Complement. Altern Med..

[79] Rao N., Kemper K.J. (2017). Online training in specific meditation practices improves gratitude, well-being, self-compassion, and confidence in providing compassionate care among health professionals. J. Evid Based Complement. Altern Med..

[80] Oliver J.J., MacLeod A.K. (2018). Working adults’ well-being: An online self-help goal-based intervention. J. Occup. Organ. Psychol..

[81] Ahtinen A., Mattila E., Välkkynen P., Kaipainen K., Vanhala T., Ermes M., Sairanen E., Myllymäki T., Lappalainen R. (2013). Mobile mental wellness training for stress management: Feasibility and design implications based on a one-month field study. JMIR mHealth uHealth.

[82] Bostock S., Crosswell A.D., Prather A.A., Steptoe A. (2019). Mindfulness on-the-go: Effects of a mindfulness meditation app on work stress and well-being. J. Occup. Health Psychol..

[83] Joyce S., Shand F., Lal T.J., Mott B., Bryant R.A., Harvey S.B. (2019). Resilience@ work mindfulness program: Results from a cluster randomized controlled trial with first responders. J. Med. Internet Res..

[84] Wylde C.M., Mahrer N.E., Meyer R.M., Gold J.I. (2017). Mindfulness for novice pediatric nurses: Smartphone application versus traditional intervention. J. Pediatr. Nurs..

[85] Querstret D., Cropley M., Fife-Schaw C. (2017). Internet-based instructor-led mindfulness for work-related rumination, fatigue, and sleep: Assessing facets of mindfulness as mechanisms of change. A randomized waitlist control trial. J. Occup. Health Psychol..

[86] Joyce S., Shand F., Bryant R.A., Lal T.J., Harvey S.B. (2018). Mindfulness-based resilience training in the workplace: Pilot study of the internet-based Resilience@ Work (RAW) mindfulness program. J. Med. Internet Res..

[87] Maunder R.G., Lancee W.J., Mae R., Vincent L., Peladeau N., Beduz M.A., Hunter J.J., Leszcz M. (2010). Computer-assisted resilience training to prepare healthcare workers for pandemic influenza: A randomized trial of the optimal dose of training. BMC Health Serv. Res..

[88] Yunus W.M., Musiat P., Brown J.S. (2019). Evaluating the feasibility of an innovative self-confidence webinar intervention for depression in the workplace: A proof-of-concept study. JMIR Ment Health.

[89] Kaplan S., Bradley-Geist J.C., Ahmad A., Anderson A., Hargrove A.K., Lindsey A. (2014). A test of two positive psychology interventions to increase employee well-being. J. Bus. Psychol..

[90] Winslow C.J., Kaplan S.A., Bradley-Geist J.C., Lindsey A.P., Ahmad A.S., Hargrove A.K. (2017). An examination of two positive organisational interventions: For whom do these interventions work?. J. Occup. Health Psychol..

[91] Phillips R., Schneider J.M., Molosankwe I., Leese M., Foroushani P.S., Grime P., McCrone P., Morriss R.K., Thornicroft G. (2014). Randomized controlled trial of computerized cognitive behavioural therapy for depressive symptoms: Effectiveness and costs of a workplace intervention. Psychol. Med..

[92] Lilly M., Calhoun R., Painter I., Beaton R., Stangenes S., Revere D., Baseman J., Meischke H. (2019). Destress 9-1-1—An online mindfulness-based intervention in reducing stress among emergency medical dispatchers: A randomised controlled trial. Occup. Environ. Med..

[93] Hasson D., Anderberg U.M., Theorell T., Arnetz B.B. (2005). Psychophysiological effects of a web-based stress management system: A prospective, randomized controlled intervention study of IT and media workers [ISRCTN54254861]. BMC Public Health.

[94] Yamagishi M., Kobayashi T., Kobayashi T., Nagami M., Shimazu A., Kageyama T. (2007). Effect of web-based assertion training for stress management of Japanese nurses. J. Nurs. Manag..

[95] Eisen K.P., Allen G.J., Bollash M., Pescatello L.S. (2008). Stress management in the workplace: A comparison of a computer-based and an in-person stress-management intervention. Comput. Hum. Behav..

[96] Gollwitzer P.M., Mayer D., Frick C., Oettingen G. (2018). Promoting the self-regulation of stress in health care providers: An internet-based intervention. Front Psychol..

[97] Williams A., Hagerty B.M., Brasington S.J., Clem J.B., Williams D.A. (2010). Stress gym: Feasibility of deploying a web-enhanced behavioral self-management program for stress in a military setting. Mil Med..

[98] Kawai K., Yamazaki Y., Nakayama K. (2010). Process evaluation of a web-based stress management program to promote psychological well-being in a sample of white-collar workers in Japan. Ind. Health.

[99] Bennett J.B., Neeper M., Linde B.D., Lucas G.M., Simone L. (2018). Team resilience training in the workplace: E-learning adaptation, measurement model, and two pilot studies. JMIR Ment. Health.

[100] Dyrbye L.N., West C.P., Richards M.L., Ross H.J., Satele D., Shanafelt T.D. (2016). A randomized, controlled study of an online intervention to promote job satisfaction and well-being among physicians. Burn Res..

[101] Abbott J.A., Klein B., Hamilton C., Rosenthal A.J. (2009). The impact of online resilience training for sales managers on wellbeing and performance. E-J Appl. Psychol..

[102] Carissoli C., Villani D., Riva G. (2015). Does a meditation protocol supported by a mobile application help people reduce stress? Suggestions from a controlled pragmatic trial. Cyberpsychol. Behav. Soc. Netw..

[103] Michie S., Prestwich A. (2010). Are interventions theory-based? Development of a theory coding scheme. Health Psychol..

[104] Karasek R.A. (1979). Job demands, job decision latitude, and mental strain: Implications for job redesign. Adm. Sci. Q..

[105] Diener E., Sapyta J.J., Suh E. (1998). Subjective well-being is essential to well-being. Psychol. Inq..

[106] Ryff C.D. (1989). Happiness is everything, or is it? Explorations on the meaning of psychological well-being. J. Pers. Soc. Psychol..

[107] Keyes C.L. (2002). The mental health continuum: From languishing to flourishing in life. J. Health Soc. Behav..

[108] Layous K., Lyubomirsky S., Gruber J., Moskowitz T.J. (2014). The how, why, what, when, and who of happiness. Positive Emotion: Integrating the Light Sides and Dark Sides.

[109] McGuinness L.A., Higgins J.P. (2021). Risk-of-bias VISualization (robvis): An R package and Shiny web app for visualizing risk-of-bias assessments. Res. Synth Methods.

[110] Donaldson S.I., Lee J.Y., Donaldson S.I. (2019). Evaluating positive psychology interventions at work: A systematic review and meta-analysis. Int. J. Appl. Posit. Psychol..

[111] Lomas T., Medina J.C., Ivtzan I., Rupprecht S., Eiroa-Orosa F.J. (2019). Mindfulness-based interventions in the workplace: An inclusive systematic review and meta-analysis of their impact upon wellbeing. J. Posit. Psychol..

[112] Knight C., Patterson M., Dawson J. (2019). Work engagement interventions can be effective: A systematic review. Eur. J. Work Organ. Psychol..

[113] Linardon J., Cuijpers P., Carlbring P., Messer M., Fuller-Tyszkiewicz M. (2019). The efficacy of app-supported smartphone interventions for mental health problems: A meta-analysis of randomized controlled trials. World Psychiatry.

[114] Hesketh R., Strang L., Pollitt A., Wilkinson B. (2020). What Do We Know about the Effectiveness of Workplace Mental Health Interventions.

[115] Bartlett L., Martin A., Neil A., Memish K., Otahal P., Kilpatrick M., Sanderson K. (2019). A systematic review and meta-analysis of workplace mindfulness training randomized controlled trials. J. Occup. Health Psychol..

[116] Ivandic I., Freeman A., Birner U., Nowak D., Sabariego C. (2017). A systematic review of brief mental health and well-being interventions in organisational settings. Scand. J. Work Environ. Health.

[117] Bellón J.Á., Conejo-Ceron S., Cortes-Abela C., Pena-Andreu J.M., Garcia-Rodriguez A., Moreno-Peral P. (2019). Effectiveness of psychological and educational interventions for the prevention of depression in the workplace: A systematic review and meta-analysis. Scand. J. Work Environ. Health.

[118] Tan L., Wang M.J., Modini M., Joyce S., Mykletun A., Christensen H., Harvey S.B. (2014). Preventing the development of depression at work: A systematic review and meta-analysis of universal interventions in the workplace. BMC Med..

[119] Taylor H., Strauss C., Cavanagh K. (2021). Can a little bit of mindfulness do you good? A systematic review and meta-analyses of unguided mindfulness-based self-help interventions. Clin. Psychol. Rev..

[120] Jarman L., Martin A., Venn A., Otahal P., Sanderson K. (2015). Does workplace health promotion contribute to job stress reduction? Three-year findings from Partnering Healthy@ Work. BMC Public Health.

[121] Kröll C., Doebler P., Nüesch S. (2017). Meta-analytic evidence of the effectiveness of stress management at work. Eur. J. Work Organ. Psychol..

[122] Stanulewicz N., Knox E., Narayanasamy M., Shivji N., Khunti K., Blake H. (2020). Effectiveness of lifestyle health promotion interventions for nurses: A systematic review. Int. J. Environ. Res. Public Health.

[123] von der Embse N., Ryan S.V., Gibbs T., Mankin A. (2019). Teacher stress interventions: A systematic review. Psychol. Sch..

[124] Rigabert A., Motrico E., Moreno-Peral P., Resurrección D.M., Conejo-Cerón S., Cuijpers P., Martín-Gómez C., López-Del-Hoyo Y., Bellón J.Á. (2020). Effectiveness of online psychological and psychoeducational interventions to prevent depression: Systematic review and meta-analysis of randomized controlled trials. Clin. Psychol. Rev..

[125] Zhang M., Murphy B., Cabanilla A., Yidi C. (2021). Physical relaxation for occupational stress in healthcare workers: A systematic review and network meta-analysis of randomized controlled trials. J. Occup. Health.

[126] Nigatu Y.T., Huang J., Rao S., Gillis K., Merali Z., Wang J. (2019). Indicated prevention interventions in the workplace for depressive symptoms: A systematic review and meta-analysis. Am. J. Prev. Med..

[127] Johns S.A., Tarver W.L., Secinti E., Mosher C.E., Stutz P.V., Carnahan J.L., Talib T.L., Shanahan M.L., Faidley M.T., Kidwell K.M. (2021). Effects of mindfulness-based interventions on fatigue in cancer survivors: A systematic review and meta-analysis of randomized controlled trials. Crit. Rev. Oncol. Hematol..

[128] Haller H., Winkler M.M., Klose P., Dobos G., Kuemmel S., Cramer H. (2017). Mindfulness-based interventions for women with breast cancer: An updated systematic review and meta-analysis. Acta Oncol..

[129] Janssen M., Heerkens Y., Kuijer W., Van Der Heijden B., Engels J. (2018). Effects of mindfulness-based stress reduction on employees’ mental health: A systematic review. PLoS ONE.

[130] Conversano C., Ciacchini R., Orrù G., Di Giuseppe M., Gemignani A., Poli A. (2020). Mindfulness, compassion, and self-compassion among health care professionals: What’s new? A systematic review. Front. Psychol..

[131] Kaminski K. (2020). Exploring compassion fatigue and burnout in healthcare professionals: A scoping review. Int. J. Nurs. Stud. Scholarsh..

[132] Hashem Z., Zeinoun P. (2020). Self-compassion explains less burnout among healthcare professionals. Mindfulness.

[133] Maricuţoiu L.P., Sava F.A., Butta O. (2016). The effectiveness of controlled interventions on employees’ burnout: A meta-analysis. J. Occup. Organ. Psychol..

[134] Fleming T., Bavin L., Lucassen M., Stasiak K., Hopkins S., Merry S. (2018). Beyond the trial: Systematic review of real-world uptake and engagement with digital self-help interventions for depression, low mood, or anxiety. J. Med. Internet Res..

[135] van Agteren J., Iasiello M., Lo L., Bartholomaeus J., Kopsaftis Z., Carey M., Kyrios M. (2021). A systematic review and meta-analysis of psychological interventions to improve mental wellbeing. Nat. Hum. Behav..

[136] Horan K.A., Streit J.M., Beltramo J.M., Post M. (2021). The application of the theory coding scheme to interventions in occupational health psychology. J. Occup. Environ. Med..

[137] Silva M.N., Marques M.M., Teixeira P.J. (2014). Testing theory in practice: The example of self-determination theory-based interventions. Eur. Health Psychol..

[138] Breslin G., Shannon S., Haughey T., Sarju N., Neill D., Leavey G., Lawlor M. (2021). Athlete and nonathlete intentions to self-manage mental health: Applying the Integrated behavior change model to the state of mind program. J. Appl. Sport Psychol..

[139] Ntoumanis N., Ng J.Y., Prestwich A., Quested E., Hancox J.E., Thøgersen-Ntoumani C., Deci E.L., Ryan R.M., Lonsdale C., Williams G.C. (2021). A meta-analysis of self-determination theory-informed intervention studies in the health domain: Effects on motivation, health behavior, physical, and psychological health. Health Psychol. Rev..

[140] Phillips E.A., Gordeev V.S., Schreyögg J. (2019). Effectiveness of occupational e-mental health interventions: A systematic review and meta-analysis of randomized controlled trials. Scand. J. Work Environ. Health.

[141] Rosenkranz M.A., Dunne J.D., Davidson R.J. (2019). The next generation of mindfulness-based intervention research: What have we learned and where are we headed?. Curr. Opin. Psychol..

