# Supplementary material for: Effectiveness of Digital Interventions for Deficit-Oriented and Asset-Oriented Psychological Outcomes in the Workplace: A Systematic Review and Narrative Synthesis
Source: Eur J Investig Health Psychol Educ. 2022 Oct 3;12(10):1471–97. doi: 10.3390/ejihpe12100102 (PMC9601105; doi:10.3390/ejihpe12100102)
Supplement: Supplementary file 1 [file ejihpe-12-00102-s001.zip › ejihpe-1887568-supplementary-revised.pdf]

Table S1: Studies details

ID	Author and Year	Settings	Intervention (s)<br>I: Intervention<br>C: Control	Participants	Method	Measures	Main findings
1	Billings et al. (2008)	-technology company in the Mid-Atlantic region (USA)	I: web-based multi-media health promotion program; self-paced audio-narrated, with ample use of video and graphics; use of cognitive-behavioural techniques; tailored to the individual user through an embedded assessment instrument<br>- access to the program for 3 months<br>-follow-up:<br>post intervention<br>C: wait-list control	309 working adults<br>M: 91<br>F: 218<br>- sample predominantly young, female, highly educated	RCT	Stress: Symptoms of Distress scale<br>Depression:<br>Center for Epidemiologic Studies Depression Scale—Revised<br>Anxiety: Beck Anxiety Inventory (BAI)<br>Negative Coping: Stress Relief Strategies questionnaire<br>Work Productivity-Limitations Questionnaire	Marginally significant reduction in stress (F = 5.23, P < 0.05<br>no other significant group differences in depression, anxiety, or mood<br>Non-significant improvement in work performance
2	Katelaar et al. (2014)	one academic hospital (Netherlands)	I:<br>A mental module for WHS, consisting of online screening on	N= 128, F: 99<br>Age; M=40<br>nurses, and allied health	Pre-post not controlled	Stress- distress subscale of the Four Dimensional Symptoms	statistically significant improvement of work functioning (t= 2.67, p < 0.01

			impaired work functioning and impaired mental health followed by personalized online feedback and online tailored advice combined with access to self-help EMH: Psyfit, Strong at work, Color your life	professionals The study participants originated from two study arms of a previous RCT		Questionnaire (4DSQ) Work-related fatigue- Dutch Questionnaire on the Experience and Evaluation of Work (QEEW) Impaired work functioning- six subscales from Nurses Work Functioning Questionnaire (NWFQ)	and work-related fatigue (t= 3.02, p < 0.01) -small meaningful effect on stress was found (Cohen d ¼ .23) among those that logged onto an EMH intervention (20%, n= 26).
3	Leary et al. (2018)	VA Healthcare System, (USA)	I: 3-month six 50-minute Internet-delivered MRP sessions, Duration: 3 months; Structure: every other week, interactive facilitated classes (about mantra repetition and slowing down as stress reduction strategy) T2: post intervention	N=39; Female: 34 Age; M= 51.21 Female: 34 postgraduate degrees (77%); nurses (36%) Did not practice meditation at baseline (n = 16)	controlled Trial	Stress of Conscience- SOC Questionnaire (SCQ) Burnout- Maslach Burnout Inventory– General Survey (MBI-GS)	beneficial effect in EX, FS, and TC between T1 and T3 and less PE and declining FS between T1 and T2 (p < 0.01)

			T3: 3 months after study completion				
4	Heeter et al. (2017)	Hospice and palliative care healthcare providers,	I: technology-assisted Yoga Therapy meditation program va smartphone apps<br><br>Structure: 10-12 minute meditations, introducing 1 meditation each week, supported by biweekly emails.<br><br>Duration: 6 weeks	N=44, Female:35<br><br>Age; M=49	Pre-post not controlled<br><br>Pilot study	Compassion fatigue and burnout- Professional Quality of life (ProQOL)<br><br>Interoceptive Awareness- Multidimensional Assessment of Interoceptive Awareness (MAIA)	Significant improvement for compassion fatigue and burnout ($p<0.05$)<br><br>Significant improvement in all MAIA subscales ($p<0.05$)
5	Joyce et al. (2019)	Cluster randomisation 24 Primary Fire and Rescue and Hazmat stations within New South Wales, Australia	I: Resilience@Work (RAW) Mindfulness Program, 6 online training sessions, combination of interactive exercises, audio, and animation<br><br>C: Healthy Living Program<br><br>6 self-paced modules on an iPad with helpful information on a range of health and well-being topics,<br><br>Duration: 3.5-6 weeks<br><br>T2:6 weeks after baseline	N=143<br><br>I: 83<br><br>(M: 56, F: 4, Age; M=43.9)<br><br>C: 60<br><br>(M: 81, F: 2, Age; M= 41.1)	Cluster RCT	-Resilience: Connor-Davidson Resilience Scale<br><br>Brief Resilience Scale<br><br>-Acceptance and Mindfulness Skills<br><br>Freiburg Mindfulness Inventory<br><br>-Cognitive Fusion Questionnaire	-significant increase in resilience over time ($p=0.01$)<br><br>- per-protocol analysis: at 6-month<br><br>a significant and positive change in CDRISC_10 resilience scores ($p=0.002$)<br><br>- statistically significant improvements for the intervention group in optimism

			T3: 6 months after baseline			-Acceptance and Action Questionnaire<br><br>Self-Compassion Scale<br><br>-Resilience Resources<br><br>Optimism: Life Orientation Test-Revised<br><br>Coping: The Brief-Coping Orientation to Problems Experienced<br><br>Sense of Purpose: Life Engagement Test	(P=.05), use of emotional support (P=.05), and use of instrumental support (P=.05) at 6-week follow-up<br><br>- significant and sustained improvement in active coping among the intervention at 6-month follow-up (P=.046)<br><br>Trend for improved cognitive fusion (p=.08) for program completers at 6-weeks
6	Shimazu et al. (2005)	Construction machinery company, Japan	I: Web-Based Psychoeducation on Self-Efficacy, Problem Solving Behaviour, Stress Responses and Job Satisfaction among Workers,<br><br>5 online chapters.<br><br>T2: 5 weeks after baseline,<br><br>T3:11 weeks after the baselines	N=255<br><br>I:112<br><br>Age;M: 41.9<br><br>C:113<br><br>Age;M: 44.0	controlled Trial	Self-efficacy- 17 items from Japanese version of Sherer and Maddux questionnaire measuring general self-efficacy (17 items) and social self-efficacy	a marginally significant<br><br>increase in job satisfaction for the intervention group from T1 to T2 (p=0.081)<br><br>no effects on job stress

						Problem solving behaviour-6 items-questionnaire developed for this study<br><br>Psychological and physical stress responses-29 items from the Brief Job Stress Questionnaire (BJSQ)<br><br>Job satisfaction-single item measure of general job satisfaction	
7	Ahtinen et al. (2013)	local technical university in Finland	I: mobile mental wellness-training app<br>stand-alone app for Android mobile phones and tablets<br>4 intervention modules or “paths”<br>3 paths teaching the 6 core processes of ACT and the fourth path focuses on physical wellness, but with	N=15, female: 8<br>university staff<br>Mean age: not mentioned	Pre-post not controll ed	Psychological flexibility-Acceptance and Action Questionnaire (AAQ-II,<br><br>The Satisfaction with Life Scale (SWLS,	statistically significant for ratings of stress (z=3.00, P=.003) and life satisfaction (z=2.32, P=.02). There was no statistically significant change in psychological flexibility (z=0.06, P=.950).

			an ACT-based approach. altogether 46 text and audio exercises.<br><br>Duration: 1 months use<br><br>T2: after 1 month use			single-item stress scale	
8	Carissoli et al. (2015)	companies and associations in Milan	I: app ‘‘It’s time to relax!’’- guided meditations; two mindfulness meditations per day, lasting 15 minutes each, by listening to the guided or free (starting from the second week) meditation<br><br>Both thought distancing and mindful breathing exercises were included,<br><br>C: brief relaxation protocol based on passive listening to relaxing music; two pieces of relaxing music (chosen from a proposed list) per day, lasting about 15 minutes each,<br><br>T2: 3 weeks after the interventions	N=56, Italian workers<br><br>I: 20<br><br>C: 18<br><br>Waiting list: 18	RCT: efficacy of a week mindfulness inspired protocol; controlled pragmatic trial	perceived stress: Italian validated version of the Mesure du Stress Psychologique (MSP) questionnaire	No statistically significant differences,

9	Dyrbye et al. (2016)	the Mayo Clinic Departments of Medicine in Minnesota and Arizona and Mayo Clinic Department of Surgery in Minnesota	I: 10 weekly surveys including a menu of 5–6 self-directed micro-tasks to select one to complete weekly<br><br>C: just the surveys<br><br>Duration: 3 months<br><br>T2: end-of-study (3 month) survey	practicing physicians (n=290)<br>M:184<br>F: 48<br><br>I: 145<br><br>C: 145	RCT	Burnout- Maslach Burnout Inventory<br><br>Symptoms of depression- Primary Care Evaluation of Mental Disorders (PRIME MD)<br><br>Quality of life (QOL)- a single-item linear<br>analog scale assessment question<br><br>Work engagement- absorption<br>sub-scale of the Work Engagement Scale<br><br>meaning in work- Empowerment at Work Scale	No statistically significant differences,
10	Gollwitzer et al. (2018	health care institutions, Germany	I1(MCII): assigned to set a goal of using MCII every	N = 129<br>Nurses	RCT	Perceived Stress- adapted version of the	Significantly less stress in the MCII group as compared to

			day for the next 3 weeks<br>I2 (IIMCII): not only assigned to set a goal of using MCII every day for the next 3 weeks but in addition to make an if-then plan in their mind<br><br>C: three questions and a personal wish with respect to achieving less stress at their work place in the<br><br>Exercises taught via e-mail<br>Duration: 3 weeks<br><br>T2: 3 weeks later	MCII:, n=41<br>modified MCII; n=41<br>Control: N=47		Perceived Stress Questionnaire-20 (PSQ-20<br><br>Work engagement- a condensed version of the Utrecht Work Engagement Scale (UWES-9	the control group (p=0.01)<br><br>Significantly more work-engagement for MCII group compared to the control group and the IIMC group (p<0.05)
11	Hersch et al. (2016	Hospitals, USA	I: web-based programme, 8 stress management modules,<br><br>C:waiting list<br><br>Duration: 3 months access	Nuses (N=104)	RCT	sources of work-<br>related stress-Nursing Stress Scale<br><br>Symptoms of distress scale<br><br>Coping with stress scale	Significant improvement for the experimental full Nursing Stress Scale (p = .00) and six of the seven subscales<br><br>No effect on work limitations questionnaire

			T2: 3 months after randomisation			Work Limitations Questionnaire (WLQ)	
12	Kim et al. (2018)	Seoul Hospitals, Korea	50-minute sessions of 1:1 therapy with 1 of 3 psychologists<br><br>I1: Mobile Videoconference-Based Intervention (adapted from the Stress Management and Resilience Training: Relaxation Response Resilience Program (SMART-3RP)<br><br>I2: In-person<br><br>C: self-care condition-educational material regarding methods to self-regulate stress (1 chapter per week)<br><br>Duration: 4 weeks<br><br>T2: post treatment<br><br>T3: 1 month post treatment	Employees (42% hospital employees) (n=81)	RCT	**Perceived stress: PSS scale**<br><br>**Resilience: Brief resilience scale**<br><br>level of emotional labor- Korean Emotional Labor Scale (KELS)<br><br>level of job stress- Korean Occupational Stress Scale (KOSS)-Short Form	significant main effects for condition on PSS (P<.001) and KOS P<.001).<br><br>The interaction between time and condition was significant for PSS, P=.03; BRS P=.008; KELS: P=.03; P=.005).<br><br>no significant differences in any clinical variable between the mobile videoconferencing condition and in-person condition at follow-up<br><br>Both conditions significant improvement in BRS scores at follow-up (p<0.05) but greater for in-

							person (p=0.001)
13	Kemper et al. (2017)	large Midwestern academic health center, USA<br><br>2 enrollment periods	I: online elective educational program in mind-body skills<br><br>Self-reflection opportunities, didactic information<br><br>about focused attention meditation, hyperlinks to peer-reviewed research,<br><br>audio recordings of guided Relaxation Response practices and links to YouTube videos	N=379<br><br>Gender: male=55<br><br>''Introduction to Stress, Resilience,<br><br>and the Relaxation Response'' module: n=357<br><br>''Clinical Effects of the Relaxation Response'': n=158<br><br>''Physiologic Effects of the Relaxation Response'': n=140	Pre-post not controlled;<br><br>prospective cohort study	Relaxation, Resilience, and Stress on a numeric rating scale ranging from 0 (not at all) to 10 (extremely).<br><br>Positive and Negative Affect Schedule.<br><br>Flourishing scale	significantly for relaxation, resilience, and stress (P < .01 for each)<br><br>significant improvement for both Positive and Negative Affect (P < .01 for both).<br><br>Significant improvement on Flourishing scale (P < .01)
14	Bolier et al. (2014)	one large academic medical centre, Netherlands<br><br>(randomisation of 58 wards)	I: workers' health surveillance module combined with personalized feedback and an offer of online interventions (OI)<br><br>C: waitlist controlled	nurses and allied health professionals (n=366)<br><br>Mean Age: 40	Cluster RCT	**positive mental health (Mental Health Continuum — Short Form, MHC-SF)**	medium-sized effect at three month follow-up and small at six month follow-up for positive mental health: Significant interaction

			Duration: minimum 6 weeks<br><br>T2: 3 months after baseline<br><br>T3:6 months after baseline	Gender:<br><br>Female: 292<br><br>I: 178<br><br>C: 188		work engagement (Utrecht Work Engagement Scale, UWES), a specific well-being measure (WHO-5 Well-being Index) and mental health symptoms (Brief Symptom Inventory, BSI)	effect of group ∗ time for positive mental health for the online group<br><br>(p = 0.03, at three months Cohen's d = 0.37) and follow-up six months follow-up (Cohen's d=0.28)<br><br>medium-sized at both time-points for the subscale psychological well-being: (Cohen's d = 0.43 at post-test and 0.50 at follow-up, F = 5.35, p = .01).<br><br>significant effect on work engagement in the OI group (p = 0.03, Cohen's d = 0.25 three months follow-up, 0.15 six months follow-up), but this seemed to be mainly due to a small deterioration in

							the control group.
15	Oliver and MacLeod (2018)	three UK Civil Service departments, UK	I: online self-help goal-setting and planning (GAP) intervention<br><br>T2: post treatment<br>Follow-up: 3 months post treatment<br><br>C: Wait-list<br>Time 2: pre treatment<br>Time 3: post treatment<br>Follow-up: 3 months	N=307<br>Gender: Female (72.9%)<br>Age bands: 35–44 (33%) or 45–54 (36%)<br><br>I:158<br>C: 149	RCT<br><br>randomized, controlled crossover design	The Positive and Negative Affect Schedule (PANAS<br><br>The Satisfaction With Life Scale<br><br>The Flourishing Scale	, GAP participants reported higher levels of PA (p=0.02) and flourishing (p=0.001).<br><br>No significant differences on life satisfaction or negative effect post intervention<br><br>Effect of the intervention over time: no control group (pre-post)<br><br>Compared to before the intervention, participants reported improved PA and flourishing directly after the intervention (p<0.001),
16	Kloos et al. (2019)	a large care organization, Netherlands	I: Online gamified multi-component	nursing staff (n=136)	Cluster RCT	General well-being: Mental Health Continuum-	general well-being and work-engagement stable (P>0.2)

		I: 2 nursing homes (n=88)<br><br>C: 2 nursing homes (n=77)	positive psychology intervention<br><br>life”; six topics of Positive Psychology: (1) positive emotions; (2) discovering and using strengths; (3) optimism; (4) self-compassion; (5) resilience, and (6) positive relations:<br><br>Structure:<br><br>psycho-education and five evidence-based positive psychology exercises<br><br>Gamified aspects: storyline of following a journey towards a flourishing life, guidance by an avatar of a professor, receiving tailored automatic feedback; training completed in chronological order<br><br>C: no intervention<br><br>Duration: 8-12 weeks; participants’ advised to complete 1 module per week	Mean Age: 42<br><br>Female: 129<br><br>I: 79<br><br>C: 49		Short Form (MHC-SF)<br><br>Job satisfaction: 5 items from the Maastricht<br><br>Job Satisfaction Scale for healthcare	and no interaction effects ($p>0.5$)<br><br>significant decline in job satisfaction for the control condition ($p<0.05$)

			T2: 12 weeks later (following the intervention)<br><br>Mandatory training				
17	Joyce et al. (2018)	Fire and Rescue New South Wales, Australia	I: Online Resilience@Work Mindfulness program<br><br>Structure: combination of psychoeducation and<br><br>mindfulness training; teaching skills and strategies drawn from evidence-based therapies; and core cognitive strategies drawn from ACT for cognitive defusion and psychological flexibility (e.g. acceptance-based emotion regulation strategies)<br><br>Duration=6 sessions	full-time firefighters (N=29)	Pre-post not controlled<br><br>Pilot study	Connor-Davidson Resilience Scale (CD-RISC 10)<br><br>(processes) Cognitive Fusion Questionnaire (CFQ) and the Acceptance and Action Questionnaire version 2 (AAQ-II)	Improvement (effect size of 0.5)<br><br>but no significant change (p=0.09)<br><br>Improvement (effect size of 1.0) in psychological flexibility but no significant change (p=.07)<br><br>No effects in cognitive fusion
18	Hirsch et al. (2017)	Firm, USA	I: myStrength<br><br>Structure: self-paced cCBT modules for depression and anxiety, mindfulness and other empirically validated tools	N= 165<br><br>I: 96<br><br>C: 69<br><br>Gender:<br><br>Female: 80	RCT<br><br>pilot	depression subscale score of the Depression, Anxiety, and Stress Scale (DASS-21)	A significant reduction in depression score was achieved at each time point (P=.002) with the experimental group having

			taught via short form video, substance use motivational interviewing and relapse prevention modules<br><br>, emails and reminders; web and mobile interface<br><br>C: weekly tip emails<br><br>Duration: 26-week<br><br>T2=14,<br><br>T3= 60<br><br>T4: 180	Male: 65			accelerated trajectory of depression symptom reduction to a factor of 1.35 times faster than the active control arm.<br><br>symptom levels began to converge by the 6-month assessment experimental group spent less time being symptomatic compared to the active control group.M=4.0, Sd=4.8 (Intervention), M=4.3, SD=5.3 (Control)
19	Rao and Kemper (2017)	Health professionals, USA (affiliated with a university)	I: 3 online meditation training modules (1 hour each):<br><br>(a) Gratitude focused Meditation, (b) Positive- or Sacred-Word-focused Meditation, and (c) Loving-kindness/Compassion-focused Meditation	diverse practitioners (nurses, physicians, social workers, and others)), n=177<br><br>Mean age=	Pre-post not controlled	Previously validated 6-item Gratitude Questionnaire<br><br>5-item World Health Organization Well-Being index<br><br>short form of Neff's	small but significant improvements in every measure—gratitude, well-being, self-compassion, and confidence in providing compassionate care (p<001)

			available scientific evidence; links to guided practices to encourage experiential learning; suggestions for incorporating each technique into clinical practice; self-reflection exercises.	Gender= Females(n= 148)		SelfCompassion Scale<br><br>the Confidence in providing Compassionate Care scale	
20	Imamura et al. (2014)	2 companies developed information technology systems and related services as their products; Japan	I: Internet CBT program: self-monitoring skills (in lesson 2), cognitive restructuring skills (in lessons 3 and 4), assertiveness (in lesson 5), problem-solving skills (in lesson 6), and relaxation skills (in lesson 4); homework; a Manga (Japanese comic) story of a psychologist and a client worker<br><br>C: **e-mail message once a month titled ''Useful information for stress management.''**<br><br>Duration: 6 weeks	Workers in two companies (n=762)<br><br>I: 381<br><br>C: 381	RCT	Beck Depression Inventory II (BDI-II)<br><br>Kessler's psychological distress scale<br><br>Japanese version of Dysfunctional Attitude Scale 24 (DAS24-J).<br><br>Self-efficacy: ''How confident are you that you can do….'' (iCBT program components)	significant effect on BDI-II ((t =-1.99, P<0.05): and DAS (t=-2.43,p=0.02), with small size effects for BDI-II<br><br>Cohen's d= -0.14 at 3 months, and Cohen's d= -0.16, at 6-months ; and for DAS -0.11 at 3 months and-0.30 at 6 months<br><br>a marginally statistically significant effect on<br><br>K6 (t=-1.72, p=0.09)<br><br>And significant effect on all

			T2: 3 months from baseline<br>T3: 6 months from baseline				efficacy variables (p<0.05) except problem solving (p=0.07).<br><br>Effects on BDI-II (d=-0.25) and DAS (d=-0.27) were stronger for participants with high psychological distress at baseline<br><br>No significant difference was observed in the remission based on BDI-II between the intervention and control groups:
21	Kawai et al. (2010)	Web access from workplace or home,	I: Web-based stress management for the promotion of psychological well-being; 4 sessions;<br>Partial CBT<br><br>Duration: 2 weeks<br><br>T2=post intervention	Employees =168<br><br>Mean Age: 39.3<br>Male: 117<br>Female: 50	Pre-post not controll ed	Ryff's Psychological well-being scale<br><br>13-item version Center for Epidemiologic Studies Depression Rating Scale (CESD)	Statistical significant improvement for psychological well-being (p<0.001)<br><br>Improvement but not significant differences for CES-D

							Participant evaluations of the sessions directly predicted changes in psychological well-being (β=0.29, p<0.01), and changes in the psychological well-being predicted changes in the CES-D (β=0.50, p<0.001)
22	Bostock et al. (2018)	One pharmaceutical and one high-tech company, UK	inviting them to a 1-hr in-person introductory talk about meditation.<br>I: a mindfulness meditation program (Headspace) delivered via a smartphone application; 45 pre-recorded 10- to 20-min guided audio meditations<br>C: waiting list<br>Duration: 45 days<br>T2: 9-11 weeks after baseline (2	Employees (n=238)<br>Mean age: 35.5<br>Female: 141<br>I: 128<br>C: 110<br>Duration: 8 weeks<br>.	RCT	Warwick Edinburgh Mental Well-being Scale<br>Daily well-being was assessed with positive emotions ratings provided five times throughout 1 working day following daily diary methodology<br>subscales of the Hospital Anxiety and	Significant improvements for the intervention group at T2<br>for well-being (F=8.77), daily positive emotions (f=8.37)<br>, anxiety symptoms (F=7.78), depression symptoms (F=15.6), job strain (F=5.39, p<.05) and especially job control (F=5.71, p<.05<br>and workplace social support

			weeks after the intervention period)<br><br>T3 (both): 8 weeks after T2 (16 weeks after baseline)			Depression Scale<br><br>Job strain was assessed with 16 items extracted<br>from the Whitehall II Study Questionnaire<br><br>Social support at work was assessed using five statements ranked on a 4-point scale anchored at strongly disagree (1) and strongly agree	(F=15.6 p< .001).<br><br>Moderate to large effects of amount of practice:<br><br>Mean<br>scores between the two time points for global well-being, depressive<br>symptoms, and job strain were not significantly different,<br>indicating that the increases in these outcomes from t1 to t2<br>remained consistent at t3, for global well-being, p>05<br>Anxiety symptom scores<br>increased slightly, t(104) 2.053, p .04.
23	Umanodan et al. (2014)	mental health promotion program	I: Computer-based psychoeducational Stress Management	12 work units	Cluster RCT	18-item<br>Brief Job Stress	no significant group × time interactions on any the r

		in a manufacturing company, Japan	training; 1) behavioural techniques, 2) communication techniques, and 3) cognitive techniques. each part was divided into cognitive behavioural skills;<br><br>a skill acquisition phase and a practice phase.<br><br>“congratulations email”,<br><br>“application enhancement email”<br><br>C: wait-list<br><br>Duration: 7-week program<br><br>T2= 19 weeks (T2) after the baseline survey.	I: 8 work units (n=142)<br><br>C: 4 work units (121)		Questionnaire (BJSQ)<br><br>short form of the Japanese version of the Utrecht Work Engagement Scale (UWES-J)<br><br>World Health<br><br>Organization (WHO) Health and Work Performance Questionnaire (HPQ)<br><br>Secondary outcomes<br><br>Brief Scales for Coping Profile (BSCP)	variables assessed (p>0.05) and no significant effects
24	Heckenberg et al. (2019)	direct-care workers employed in regional Victoria, Australia	I: Online Mindfulness-<br><br>Based Stress Reduction program (website): weekly reading and videos as well as 30 min sessions of formal mindfulness meditation six days per week.	n=22<br><br>Mean age: M=43.2	Pre-post not controlled	short-term psychological states:<br><br>Two subscales from the Profile of Mood States (POMS	Significant improvement for state anxiety (t=2.90, p<0.05),<br><br>Fatigue t=2.76, p=.005

			mood and state anxiety measures: before and immediately after the first prescribed formal practice of the intervention in weeks one, four and seven<br><br>Duration: 8 weeks			vigor<br>and fatigue (12 items) and the 6-item Spielberger State-trait Anxiety Index (STAI;<br><br>Enduring psychological states:<br><br>Effort reward imbalance<br>Questionnaire<br><br>Over-commitment was assessed using a 6-item scale assessing in-<br>ability to withdraw from work, impatience, and disproportionate irritability<br><br>the 10-item PSS<br><br>4-item Freiberg Mindfulness	(controlled for active participation)<br><br>Mindfulness (t=4.03, p<0.001),<br>Overcommitment (t=2.46, p<0.05), and<br>Optimism (t=3.16, p<0.05)<br><br>Work engagement also approached significance<br>(p=.07)

						Inventory - Short Form (FMI)<br><br>9-item subscale from the<br>Job Demands-Resources Questionnaire<br><br>Two 4-item subscales from the Job Demands-Resources Questionnaire<br><br>6-item Brief Resilience Scale	
25	Wan Mohd Yunus et al. (2019)	2 companies (administrative body and a local authority), UK	I: webinar, six sessions: Web-based seminars ; computer, laptop, or mobile device or tablets;<br>a PowerPoint presentation with videos and comic strips, an Interactive Zones component where participants interacted with each other, a	Employees (n=33)<br><br>Mean age: 39.6<br>Gender: Female (n=30), Male (n=3)	Pre-post not controlled	Beck Anxiety Inventory<br>Beck Depression Inventory<br><br>Rosenberg Self-Esteem Scale<br><br>short form World Health Organization	Significant effect over time for depression (F=31.5; P<.001), anxiety (F=3.9; P=.03), self-esteem (F=31.524; P=.02), and coping flexibility (F=14.184; P<.001).<br><br>Statistical significant

			virtual whiteboard, use of the chat feature, and a webcam feature for the therapist<br><br>cognitive-behavioural approach by Brown et al<br><br>and coping flexibility by Cheng<br><br>Duration: 6 weeks<br><br>T2: Post intervention<br><br>T3: 1 months post intervention			Health and Work Performance Questionnaire	differences both Absolute presenteeism (x2=22.1)<br><br>And relative presenteeism (x2=26.3), p's<.001 for the study period.<br><br>highest effect size recorded for presenteeism measures (Hedges gav=-.96), followed by coping flexibility (Hedges gav=–0.81) and depression (Hedges gav= 0.52)<br><br>Not statistical significant effects for absenteeism
26	Imamura et al. (2015)	two information technology companies, Japan<br><br>Duration: 6 weeks	I: iCBT program: web-based training course consisting of weekly 30-minute training sessions in CBT-based stress management skills: elf-monitoring (in lesson 2), cognitive	Workers (n=762)<br><br>I: 381<br><br>C: 381<br><br>Males: 641	RCT	(secondary outcomes)<br><br>UWES<br><br>one item from the WHO Health and	a significant effect on the UWES (t = 2.03, P= 0.04). Those effect sizes were small: 0.11 at 3-month follow-up and 0.16 at 6-month follow-up.

			restructuring (in lesson 3), cognitive restructuring and relaxation (in lesson 4), assertiveness (in lesson 5), and problem solving (in lesson 6).<br><br>C: a short email message of non-CBT stress management tips<br><br>(both could use an internal employee assistance program service and both received one-session e-learning program for stress management during the study<br><br>T2: 3 MONTHS<br><br>T3: 6 months			Work Performance Questionnaire (HPQ).<br><br>Beck Depression inventory (mediator)<br><br>Sick leave: self-reported for last 3 months	Marginally statistically significant effect on sick leave days during past 3 months (t =−1.84, P = 0.07) ,<br><br>marginally significant indirect effects on work-engagement through changes in depression (p's<0.1)<br><br>No significant program effect on work performance
27	Baek et al. (2018)	Seoul National University Bundang Hospital (SNUBH) health promotion centre and large public enterprises, Korea	I: a fully automated smartphone-based stress management application<br><br>Content (among others)<br><br>-Mental health and lifestyle assessment part:	Healthy employees (N=68)<br><br>2013 sample (N=30)<br><br>Age mean: 34.8	Pre-post not controlled	Korean version of the brief encounter psychosocial instrument (BEPSI-K)<br><br>Epidemiologic studies	Significant reduction in BEPSI-K in 2013 (x2=12.49, p=0.001) and in 2014 (x2=17.15, p<0.001); for both males and females as well as younger and older

			personalised recommendations, psychoeducation and cognitive-behavioural techniques to modify their maladaptive thoughts and behaviours.<br><br>-Daily mood with life style monitoring<br><br>-Relaxation training part (only in 2014 sample)<br><br>Duration: 4 weeks<br><br>Time 2: after the intervention	Gender: Male(n=19)<br><br>Female (n=11)<br><br>2014 sample (N=38)<br><br>Age mean: 36.4<br><br>Gender: Male (n=9), Female (n=29)		depression scale (CES-D)<br><br>Others:<br><br>social readjustment rating scale (SRRS)<br><br>Framingham type A behaviour pattern (FTA)<br><br>Korean occupational stress scale (KOSS) brief version<br><br>Korean resilience quotient-53 (KRQ-53)	participants (p's<0.001)<br><br>And for the depression scale only in 2014 (x2=9.75, p=0.004)<br><br>Only BEPSI-K showed satisfactory power in 2014 at α=(0.05/9).<br><br>Females showed a significant reduction in CES-D (F=7.86, p<01) and (FTA (F=5.04, P<0.05)<br><br>Younger participants (<35 ys, N=34) showed a reduction in CES-D (F= 10.36, P=0.003);<br><br>Older participants showed a reduction in KOSS scores (F= 7.08, P= 0.01)<br><br>No effects on resilience
28	Feicht et al. (2013)	2 departments of a local insurance company	I: online training focusing on exercises for	German-speaking,	RCT<br><br>pilot	Visual Analog Scale (VAS),	At Time 2 significant differences for

			achieving a positive psychological state. In addition to an introductory week and a final week, there are 5 weeks each with three exercises for 1 happiness-relevant<br><br>C: waiting list<br><br>partly at work and partly at home<br><br>Duration: 7 weeks<br><br>T2: post intervention<br><br>T3: 4 weeks after intervention	Employees (N= 147)<br><br>I: 85		WHO-Five Well-being Index (WHO-5)<br><br>Stress Warning Signals Scale (SWS)<br><br>Secondary: Freiburg Mindfulness Inventory (FMI)<br><br>The Recovery Experience Questionnaire<br><br>The Flourishing Scale (FS)	happiness (U =603, P = 0.000), satisfaction (U=532, p<0.001), quality of life (U=624, P<0.001) and stress warning, particularly emotional stress (U = 757, P <0.001); effect sizes for satisfaction and quality of life was>1.<br><br>Strong positive effects on mindfulness (U = 866, P= 0.006), recovery experience (U = 824.5, P = 0.002, , and flourishing (U = 951, P = 0.03).<br><br>At T3 significant differences for happiness (U=681) , satisfaction (U=588.5), quality of life (U=664.5), Stress Warning Signals (U=741.5) P's<0.001, with large effect sizes (d>.80)<br><br>At time 3 no significant

							differences for Recovery experience and flourishing.
29	Crivelli et al. (2019)	a public service company, Italy	attention on breathing and related bodily sensations- mindfulness practice and a wearable neurofeedback system managed via smartphone<br><br>daily sessions of practice (total number of<br><br>sessions: 14) from 10min a day till 20min a day<br><br>Duration: 2 weeks<br><br>T2=post training	Professionals with top management duties (N=16)	Pre-post not controlled	PSS<br><br>state subscale of the STAI (situational anxiety)<br><br>Profile of Mood States (POMS) inventory	significant decrease of perceived stress scores (t=−2.341, p=0.03, situational anxiety (STAI-state subscale, t=−3.640, p=0.002, and both anger t=−5.882, p=0.001<br><br>and fatigue scores of the POMS inventory (fatigue: t=−3.878, p= 0.001<br><br>no statistical significant changes for the other POMS sub-scales
30	Wood et al. (2017)	VA Puget Sound Health Care System, USA	I: Provider Resilience (PR) mobile application: two assessment tools to help increase self-awareness of current levels of burnout: the Professional Quality of Life Scale (ProQOL) and the Burnout Visual	Outpatient mental health providers (N = 30)<br><br>Age (M = 42.5)<br><br>Gender (no information)	Pre-post not controlled<br><br>Pilot study	Professional Quality of Life-Revision IV (ProQOL<br><br>Additional:<br><br>Outcome<br><br>Questionnaire 45 (OQ-45;	significant decreases in both the Burnout (t=3.65) and the Compassion Fatigue/ Secondary Trauma subscale (t= 4.54) p’s<0.001

			Analog Scale. The home screen provides an overall graphic of the user's current resilience rating, and customizable resilience "Builders" and "Killers", which encourages users to be aware of factors that both increase and decrease their resilience<br><br>Duration: 1 month<br>T2: post treatment			Connor-Davidson Resilience Scale (CD-RISC	No significant changes for compassion satisfaction<br>Interpersonal Relations subscale not significant after Bonferroni corrections
31	Bennett et al. (2018)	Firms within a national engineering association through "Wellness Champions" within their firm, USA	I: Team resilience-Web-based training; access via desktop<br><br>e-learning module (55 slides) on participants' ability to be resilient in the workplace, their knowledge of resiliency, their awareness of resources, and their willingness to access those resources when necessary.<br><br>C(sample 2): email asking for input from those who	Employees in engineering firms,<br>Sample 1=118<br>Gender: (Male: 75)<br><br>Sample 2: (I: 118, C: 201)<br>Gender (M=174)<br>Mean age: no information	Only post study<br><br>2 pilot-studies (one not controlled and one not controlled)	3 items derived from a review of recent writings on the topic of perceived workplace resilience<br><br>4 items adapted from the Connor-Davidson Resilience Scale (for dispositional resilience)	Significant effect was for perceived workplace resilience (t= 11.44, p<0.001)<br><br>No effects for dispositional resilience

			had never completed any wellness program.<br><br>Duration: 4-6 weeks<br>T2: post training				
32	Eisen et al. (2008)	three manufacturing sites of a corporation, USA	I: completing a two-session cognitive-behavioural stress-management intervention on-line from their own computer<br>Terminal<br>1 week: 4 modules<br>2 week: 4 modules<br><br>Each module was followed by a two-minute mini-relaxation t<br><br>I2: completing the identical intervention as a two-session instructor led workshop<br><br>C: waiting list	Employees (n=289)<br>I: 123<br>I2: 134<br>C: 31	RCT	emotional health domain of the Johnson and Johnson Health Care System Insight + Health Risk Appraisal survey<br><br>Four items related to specific stressors in the workplace were selected from the Occupational Stress Inventory – Revised Edition (OSI-R<br><br>Subjective Units of Distress (SUDS)	in both groups reported a highly significant decrease in SUDS from the<br>beginning to the end of each session of the intervention ($F(1,41) = 64.52$, $p < 0.001$).<br><br>a significantly greater reduction in SUDS in Session 2 than in Session 1 ($F(1,41) = 8.44$, $p < 0.01$), suggesting that the mini-relaxation grew more effective with practice. Finally, it was found that individuals assigned to the IP group experienced a

			duration: 2 weeks<br><br>T2: post intervention<br><br>T3: 1 month follow-up				greater reduction in SUDS during both sessions than did individuals assigned to the CB group (F(1,41) = 4.45, p < 0.05).
33	Lilly et al. (2019)	9-1-1 call centres, USA and Canada	I: online mindfulness, training, adapted MBSR<br><br>7 modules (20 to 30 min to complete each), two emails each week (one introductory, one practice reminder): a short introductory video, text describing themes and activities, an audio-guided meditation exercise, suggestions for daily mindfulness activities and a moderated discussion board<br><br>C: waitlist<br><br>Duration: 7 weeks<br><br>T2: post intervention<br><br>T3:3 months post intervention	active-duty 9-1-1 telecommunicators (n=323)<br><br>I: 163<br><br>C:160<br><br>Mean Age: no information<br><br>Gender:<br><br>Male: 58<br><br>Female: 265	RCT	The Calgary Symptoms of Stress Inventory (C-SOSI)<br><br>Secondary outcome:<br><br>The Mindfulness Scale (The Mindful Attention Awareness Scale (MAAS)	significant reductions in stress scores (T2 change from baseline: M=-7.2, p<0.001), T3 change from baseline: M=-5.0, P=0.030)<br><br>with pre–post effect sizes in the small to moderate range (Cohen’s d=0.34)<br><br>at 3 months follow-up intervention participants continued to show reduced stress scores (Cohen’s d=0.22).<br><br>Not statistical significant differences for mindfulness

34	Villani et al. (2013)	day-hospital medical oncology wards, Italy	I: Mobile Stress Inoculation Training, , eight video clips with a narrative (6 showing a relaxing virtual environment and 2 presenting oncology patients suffering from cancer)<br><br>C: eight neutral video clips<br><br>Duration: 4 weeks<br>Post measures after each session	Mesure du Stress Psychologique: select nurses with a cut-off level of stress corresponding to the higher quartile level of stress<br><br>Female oncology nurses (n=30)<br>Mean age: 43<br><br>I: 15<br>C: 15	RCT	State Trait Anxiety Inventory<br>Italian translation of four items of the Brief Coping Orientation to Problems Experienced (COPE) questionnaire(2 questions)<br>Job Content Questionnaire (JCQ)	a significant decrease in anxiety (t=2.26, p<.05), and denial (t=2.91, p<.05) and increase in active coping (t=-2.46, p<.05) for the intervention group
35	Wylde et al. (2017)	RN residency program, USA	I1: Smartphone Delivered Mindfulness: 3-month sub-<br>scription to the Headspace application<br><br>I2: Traditionally Delivered Mindfulness: 1<br>session per week for 4 weeks led by a trained Buddhist Priest in the morning prior to	Novice nurses (n=96)<br><br>I1: 46<br>I2: 49<br><br>Mean Age: no information	Pre-post not controlled	The Compassion Fatigue Self Test (only T2)<br><br>Five Facet Mind-<br>fulness Questionnaire (FFMQ<br><br>The Life Events Checklist (LEC)	[TDM group predicted more compassion fatigue compared to the SDM group (b = 36.45, p = 0.05) but only if PTSD symptoms were below the clinical cut-off]<br>Marginal differences (p<.10) in compassion satisfaction and burnout with

			the residency training.<br><br>Duration: one time per week, 4 weeks<br><br>T2: post training	Gender: no informatio n		Post-traumatic stress disorder Checklist-Civilian Version (PCL-C	the TDM group predicting marginally less compassion satisfaction (b =−4.58, p b 0.10) and more burnout (b = 3.94, p b 0.10).<br><br>The TDM group showed significantly lower levels of “acting with awareness skills” (b=−2.309, p< 0.01). and marginally less non-reactivity to inner experience to the SDM group (b = −1.31, p< 0.10)
36	Williams et al. (2010)	Naval Medical Center, Portsmouth, Virginia, USA	I: a web-enhanced behavioural self-management program (WEB-SM) entitled “Stress Gym: 7 modules focusing on managing stress by developing an awareness of stress in interpersonal relationships, stress associated with dealing with specific health issues, and maladaptive responses to stress	officers and enlisted sailors (n=142)<br><br>Officers (n=27<br><br>(Mean age: 41.1)<br><br>Enlisted sailors (Mean age: 29.5)<br><br>Gender: Males: 45	Pre-post not controll ed	Single analogue scale	Significant differences were noted for both officers (t=2.12 (p.004<br><br>and enlisted members (t=5.80, p=0.0001 using paired t -test before and after using Stress Gym Data from this study

				Females: 78			
37	Hasson et al. (2005)	four information technology and two media companies, Sweden	I: web-based health promotion and stress management training: real-time daily monitoring (20-40 sec) of perceived current health and stress status, a diary and information about stress and health; web-based cognitive exercises(1-60 min): techniques for relaxation, time management, cognitive reframing and a chat<br><br>C: reference group<br><br>*(The only thing that distinguished the groups was the addition of the interactive cognitive exercises and a chat for the intervention group*)<br><br>Duration:6 months	Employees in 22 departments/units (n=303)<br><br>I: 12 departments/units (n=129)<br><br>C: 10 departments/units (n=174)	RCT	Visual analogue scales	Significant improvement for the intervention group on ratings of perceived ability to manage stress, mental energy, concentration ability, social support and competence usage at work (2way ANCOVA, $p < .05$ time × group effect).<br><br>With the exception for competence usage at work, differences between the groups remained significant when applying the non-parametric Mann-Whitney U test ($p < .05$, two-tailed).<br><br>Intervention group significantly predicted

			T2: Post intervention				improvement or beneficial changes in stress management (OR 2.3640 mental energy (OR 2.194) and social support (OR 2.752)<br><br>all participants received some kind of intervention. there were several health-related statistically significant improvements for both groups over time (time effect) such as self-esteem and work joy improved. No passive group so it is not certain that these effects can be attributed to the web-based tool
38	Phillips et al. (2014)	Occupational health sections of three large employers, UK	I: computerized CBT intervention (MoodGYM)- five, 1 h-long modules, usually taken weekly	Employees (n=organization A: 396, B: 100, and C: 141)	RCT<br><br>a pragmatic trial	WSAS: Work and Social Adjustment Scale<br><br>PHQ-9<br><br>Clinical Outcomes in	no evidence of a difference in effect between MoodGYM and control.

			C: 'attentional' control (five websites with general information about mental health).<br><br>Duration: 5 weeks<br><br>T2: post intervention<br><br>T3: 12 weeks post baseline	on the Patient Health Questionnaire-9 scored 2 or more on five of the nine items, including 2 or more on item 1 (little interest in doing things) or item 2 (feeling hopeless). and at least one of the items identified as a problem for them made it difficult to work, take care of things at home, or get along with other people<br><br>I: 318<br><br>Gender: Male:136<br><br>Female: 176		Routine Evaluation<br><br>five-domain EuroQol (EQ-5D<br><br>generalized anxiety disorder<br><br>self-assessed absence<br><br>from work<br><br>Service use [Client Service Receipt Inventory (CSRI); and quality of life (EQ-5D)	

				Mean age: 42.7<br>C: 319<br>Gender: Male: 160<br>Female: 152<br>Mean age: 42.2			
39	Kaplan et al. (2014)	two large public universities, USA	I: Gratitude condition- completed intervention online logging in at least three times per week to record things that they are grateful for related to their job<br><br>I2: Social connectedness- engage in specific strategies to increase their social ties at work social three times per week and to document those experiences on a secure Web site<br><br>For both: e-mail slide presentation with instructions, weekly<br><br>Duration: 3 days per week, 2 weeks	University employees (n=67)<br><br>Mean age: 42.9<br><br>Gender (percentages only)<br><br>I1: N=33<br>I2: N=34	Randomised trial	Primary:<br>three-item gratitude adjective checklist (GAC)<br>four items from the social connectedness subscale from Lee and Robbins' (1995) measure of belongingness.<br>PAWB and NAWB were measured with the Job-Related Affective Well-being Scale (JAWS)<br>Secondary:<br>Self-reported absence due to illness	no significant effect of the social connectedness intervention on gratitude (p>.10)<br><br>the social connectedness exercise did not significantly improve PAWB or self-reported social connectedness or gratitude.(p>.10)<br><br>Self-reported gratitude only increased for those in the gratitude condition (t=2.62, p<.05)<br><br>PAWB only increased for those in the

			T2: post intervention<br><br>T3: 4 weeks after intervention				gratitude group (p<.05)<br><br>social connectedness as the outcome revealed that this variable did not significantly increase over time across condition and did not change more dramatically for one condition versus the other (p>.10)<br><br>“ The current results indicate that gratitude did not, in fact, mediate the relationship between the gratitude intervention and PAWB. Future researchers may wish to explore other theoretical mechanisms mentioned previously (e.g., reducing cognitive dissonance and preventing ‘‘hedonic treadmill’’

40	Querstret et al. (2017)	organisations with which the university had relationships to request they promote the study to their staff and social media	I: internet-based instructor led mindfulness-online mindfulness course comprising elements of MBSR and mindfulness-based cognitive therapy (instructional videos that guide formal meditations through a website)<br><br>C: waitlist<br><br>Duration: 4 weeks<br>T2: post treatment<br><br>T3: 3 months post treatment<br><br>T4: 6 months post treatment	Employees (n=118)<br><br>I: 60<br>C: 58<br><br>Gender: Female: 95<br>Mean age: 40.68	RCT	The WRRQ (Cropley et al., 2012). Two of the WRRQ subscales—Affective Rumination and Problem-Solving Pondering<br><br>The Occupational Fatigue Exhaustion Recovery scale<br><br>Five facet mindfulness (mediation variables)	significant effect of the intervention on affective rumination, F=13.75, p < .001, problem-solving pondering,<br>F=16.01, p<001, chronic fatigue, F=33.70, p<.001, and acute fatigue F=<br>30.79, p< .001<br><br>a significant effect of the intervention on acting with awareness, F= 42.94, p<0.001, describing, F=5.76, p = .02, and nonjudging, F=26.13, p < .001,<br>the intervention did<br>not affect the nonreacting facet of mindfulness, p >.10<br><br>only acting with awareness operated as a mediator for the effect of the intervention on

							affective rumination, chronic fatigue, and acute fatigue, p’s<.01<br><br>for both groups, there was a significant main effect of the mindfulness intervention over time for all of the outcome variables (p<0.001)
41	Winslow et al. (2017)	a large social services agency, USA	I1: gratitude intervention: twice weekly, participants<br>were asked to think about and record two things in their job or work for which they are grateful<br><br>I2: gratitude and social connectedness intervention: participants were asked to engage in one of several activities and gratitude intervention<br><br>For both: a link to a secure website in	Employees (n=92)<br>I1: 28<br>I2:25<br>C: 39<br><br>Mean age: 46.63	controlled	Primary outcomes:<br>abbreviated version of the Job-Related Affective Well-Being Scale<br>Additional outcomes:<br>Gratitude Adjective Checklist<br><br>four items selected from the Social Connectedness scale measuring belongingness<br><br>Job satisfaction-	no significant mean differences between the groups<br><br>intervention assignment did not significantly predict increased gratitude for the gratitude group; or increased social connectedness for the mixed group. intervention group assignment did not significantly predict increased PAWB decreased

			which participants anonymously logged in using their unique personal identifier code and recorded activity completion<br><br>C: waitlist<br><br>Duration: 4 weeks<br><br>TW: post intervention			four items adapted from a measure developed by Brayfield and Rothe (1951)<br><br>Turnover intentions- using two items from a scale developed by Cammann, Fichman, Jenkins, and Klesh (1983	NAWB or job stress, p’s>.10
42	Abbott et al. (2009)	an industrial organisation, Australia	I: internet-based ResilienceOnline (ROL) program: Resilience Factor Inventory and graphical feedback, learning seven skills<br>of resilience, a conference call<br><br>C: waitlist<br><br>Duration: 10 weeks<br>T2:post intervention	Sales managers based in home offices (n=53)<br>I: 26<br>C: 27	RCT	Authentic<br>Happiness Inventory<br><br>The World Health Organization Quality of Life – BREF<br><br>Depression Anxiety and Stress Scales (DASS-21)<br>Statistics for individual work performance (i.e.,	No significant effects for any outcome

						margin of product sold, and volume of product sold) were obtained from the organisation for the months of June 2008 and October 2008,	
4 3	Ly et al. (2014)	medium- and large-sized companies, Sweden	I: ACT-based stress intervention: smartphone-based intervention- 6 modules: step-by-step behaviour program with the purpose of educating the participant to use ACT's six basic principles to handle their stress, small exercises (e.g. Actively explore the feeling of stress in your body), short text messages to the participants via a messaging system,<br><br>C: waiting list<br><br>Duration: 6 weeks<br><br>T2: Post intervention	Middle managers (n=72)<br><br>I: 36<br><br>C:37<br><br>Age Mean: 41.5<br><br>Gender	RCT	General Health Questionnaire (GHQ-12;<br><br>Pre, post and weekly basis<br><br>Perceived Stress Scale (PSS-14)<br><br>Multifactor Leadership<br><br>Questionnaire	Intervention group rated significantly lower their health (F=6.77; p = .010) and stress levels (F= 7.67; p =.007.<br><br>Significant (p<01) interaction effect of group and time: within-group effects for GHQ-12 (d = 0.41) an PSS-14 (d=.50) and within group effects for the intervention group GHQ-12 (d = 0.37) and PSS-14 (d= 0.62

							no effect on transformative leadership
44	Maunder et al. (2010)	a teaching hospital, Canada	I1:short (7 sessions, median cumulative duration 111 minutes)<br><br>I2: medium (12 sessions, 158 minutes)<br><br>I3: long (17 sessions, duration: 223 minutes)<br><br>Internet-based training Course: Pandemic Influenza Stress Vaccine-<br><br>Knowledge-based modules, Relaxation skills through audio modules guiding participants in progressive muscle relaxation, relaxation breathing, imagery, and combined techniques; Interactive reflective exercises<br><br>T2: post treatment	Hospital workers (n=158)	Randomised trial	Pandemic Self-Efficacy Scale (a questionnaire derived to measure responses of healthcare workers to SARS modified to apply to influenza)<br><br>Inventory of Interpersonal Problems<br><br>Ways of Coping Inventory	each course length resulted in improved self-efficacy (p<0.001)<br><br>Total interpersonal problems decreased for participants of the medium (p= 001) and long courses (p<.001) but not for those who took the short course.<br><br>medium length course sufficient for significant improvements in pandemic self-efficacy, and interpersonal problems (p=0.001)<br><br>No significance changes for

							ways of Coping with stress using problem-solving, seeking support from others or through escape-avoidance Only for those that under-utilized those strategies (score<1.5) there was a significant improvement (p's<.01)
45	Grime (2004)	a London NHS occupational health department, UK	I: computerized cognitive behavioural therapy<br><br>programme, 'Beating The Blues': cognitive and behavioural components<br><br>plus<br><br>Conventional care;<br><br>Programme In a stand-alone<br><br>computer in a private room in the Occupational Health Department.<br><br>C: conventional care<br><br>Duration: 8 weeks	NHS and local authority employees<br><br>With ten or more cumulative days of sickness absence due to stress, anxiety or depression in the past 6 months, and scored 4 or more on the GHQ-12 (General Health Questionnaire)<br><br>I: 24	RCT	Hospital Anxiety and Depression Scale (HADS<br><br>Attributional Style Questionnaire	At end of treatment, significant reduction in mean scores (adjusted for baselines scores and sex ratio) for depression (-3.07), p=.02, and negative attributional style scores (-2.32) , p=.01 in the intervention group<br><br>One month post-treatment, significant reduction in anxiety scores (-3.19) p=.02, depression scores (-2.72), p=.04, and negative attributional style (-1.95), p=.04 in the

			T2: post treatment<br><br>T3:1 month<br>T4: 3 months post treatment<br>T5: 6 months treatment	Mean age: 41<br>C:24<br>Mean age: 37			intervention group.<br><br>No effect on 3 and 6 months follow-up
46	Yamagishi et al. (2007)	urban tertiary hospital, Japan	I: 70 minutes web-based assertion training programme; content related to the definition of assertion, types of self-expression and methods for improving one's assertion ability. Learning styles: text-based learning, checklists for self-expression and the selection of suitable behaviours depending on the situation<br><br>Duration: completed over 3 weeks (workplace or home)<br>T2: post training	14 wards, Nurses (n=32)<br>(26 completed)<br>(25 analysed)<br><br>Mean age: 32.7	Pre-post not controlled	Assertive attitudes- Assertive Mind Scale (AMS, 20 items)<br><br>assertive behaviours- Assertion Check List (ACL, 20 items)<br><br>job Stress Brief Questionnaire (20 items;<br>depression- a part of the Brief Job Stress Questionnaire (18 items	Voluntary behaviour of ACL increased significantly at post-training (=+.14, p=.01) and remained significantly high 1 month later (=+.12, p=.03)<br>Participants with low scores (scores divided by the median of each assertion subscale) improvement in respect for others at post-training and 1 month late (p's<.05); and rational belief at post-training (p<.001)<br>No significant changes in job

			T3: 1 month after training					stress and depression
47	Geraedts et al. (2014a)	6 different companies, Netherlands	I: Web-based guided self-help intervention: 2 evidence-based treatments; problem-solving treatment (PST) And cognitive therapy, guideline for employees to help them to prevent work-related stress<br>6 weekly lessons<br>Duration: 6 weeks<br>T2: post intervention	Employees (n=231)<br>I: 116<br>C: 115<br>Gender: Female (N= 144)<br>Mean Age: 43.4	RCT	Center for Epidemiologic Studies Depression scale (CES-D)<br>Maslach Burnout Inventory-General Scale<br>Work absenteeism- the second part of the Trimbos and iMTA Questionnaire on Costs Associated with Psychiatric Illness (TiC-P) the Short Form Health and Labor Questionnaire (SF-HLQ<br>Work performance- the general work performance scale of the World Health Organization (WHO) Health and Work Performance Questionnaire	No significant effects

48	Geraedts et al. (2014b)	6 different (international) companies, Netherlands	I: Web-based guided self-help course for employees with depressive symptoms; 6 weekly lessons; feedback on the assignments and motivational and empathic strategies; Themes of the lessons are introduction of problem solving (lesson 1), problem-solving methods (lesson 2), changing cognitions (lesson 3), dealing with work-related problems (lesson 4), social support (lesson 5), and relapse prevention (lesson 6).<br><br>C: care as usual<br><br>Duration= 5 weeks	employees =231,<br><br>elevated depressive symptoms as measured by a score of 16 or higher on the Center for Epidemiologic Studies Depression scale (CES-D), were not on partial or full sick leave<br><br>I: 116<br><br>C: 115<br><br>Gender: Female (N= 144)<br><br>Male (n= 87<br><br>Mean Age: 43.4	RCT	Primary outcome<br><br>Center for Epidemiological Studies Depression scale (CES-D)<br><br>Secondary outcomes<br><br>Hospital Anxiety and Depression Scale (HADS)<br><br>Maslach Burnout Inventory-General Scale (MBI)<br><br>World Health Organization (WHO) Health and Work Performance Questionnaire (HPQ)	Not significant differences for primary outcome<br><br>Statistically significant between-group effects in favour of the intervention were found for anxiety symptoms (d=.16) and the exhaustion dimension of the MBI (d=.17), but with small effect sizes p's<.001<br><br>No additional gains of the intervention on the other outcomes cynicism, reduced professional efficacy, and work performance
49	Ebert et al. (2014)	educational sector, Germany	I: internet-based problem-solving training (iPST): five lessons on problem-solving techniques; behavioural	Teachers with depressive symptoms ((with a score of ≥16 on the	RCT	Primary outcome: CES-D<br><br>Secondary	Significant greater reduction in depressive symptoms severity for the intervention

			activation with respect to important values in life.<br>Plus techniques for coping with rumination, video introductions for each lesson, and example teacher characters who depict the targeted problems and demonstrate implementation of problem solving techniques<br>Within 48 hours, participants received personalized written feedback from an eCoach on the exercises they had complete<br><br>C: waitlist<br><br>Duration: 5 weeks (one lesson per week)<br><br>T2: post-intervention (7 weeks after randomisation<br><br>T3: 3 months after randomisation	Center for Epidemiologic Studies Depression Scale)<br>(n=150)<br>I: 175<br>C: 75<br><br>Mean Age: 47.1<br><br>Gender:<br>Female (n=125)		General Self-Efficacy Scale (GSE)<br>Maslach Burnout Inventory<br>Perceived Stress Questionnaire (PSQ)<br>Penn State Worry Questionnaire (PSWQ)<br>SF-12 Health Survey<br>self-reported sick leave of participants	group over time and was maintained in the follow-ups (p<.01).<br>The intervention group showed significant reliable improvement in depressive symptom severity:<br>seven weeks after randomisation (iPST: N=37 [49.3%], WLC: N=16 [21.3%], P<0.01), (ii) three months after the treatment (iPST: N=35 [46.7%], WLC: N=25 [33.3%], P=0.10), and (iii) six months after the treatment (iPST: N=34 [45.3%], WLC: N=17 [22.7%], P<0.01).<br>Significantly more intervention group participants reached symptom free status at 7 weeks (p<.01), after 3 months

			T4: 6 months after randomisation					(p=.02) and after 6 months (p=.07)<br><br>Secondary outcomes:<br><br>Significant (p<.001-p<.10) moderate effects at the intervention group for perceived stress , general self-efficacy and worrying at 7 weeks and at 2 months; with small to moderate effects at 6 months.<br><br>Moderate post treatment effect sizes for secondary outcomes with significant interactions for perceived stress (d=.36), generalised self-efficacy (d=0.47) and worrying (d=.63)<br><br>Moderate 3 months effect for perceived stress (d=.28), GSE (d=.38) and worrying (d=.54)

							At the six-month small to moderate effects for perceived stress (d=.36), GSE (D=.40) and worrying (d=0.54)(95% CI 0.21–0.68) for worrying.<br><br>No significant interactions for MBI and physical health scales.<br><br>Nos significant differences in absenteeism.
50	Coelhoso et al. (2019)	private tertiary care hospital, Brazil	I: well-being mobile app: relaxation training, breathing techniques, meditation, and positive psychology principles –<br><br>theoretical content about physical and psychological consequences of chronic stress and the use of breathing techniques; body scan; mindful breathing to	Female hospital staff (N=490)<br><br>I: 250<br><br>C:240<br><br>Mean age: 34.6	RCT	Primary outcomes<br><br>version of the 10-item perceived stress scale (PSS-10)<br><br>the Brazilian Portuguese version of the 5-item World Health Organization Well-Being Index (WHO-5)	Significant time x group interactions showed that at mid-intervention the intervention group experienced significant decrease in perceived stress (F=9.48, p=.002), increase on the well-being index (F=8.54, p=.004), greater reduction in

			cultivate present moment awareness and meditation; information on cultivating positive emotions and its impact on one's health, life, and interpersonal relationships; theoretical content focused on the influence of positive and negative thoughts on different emotional states, and a guided exercise; concept of empathy and cultivation of gratitude and guided exercises<br><br>C: monitoring of perceptions app (self-observation and evaluation of subjective levels of stress and well-being)<br><br>Duration: 8-week program with 4 lessons per week<br><br>T2: 4 weeks after randomisation (first 4 weeks of intervention			Sliding percentage of subjective Symptoms of Stress and Well-Being During the Last Month<br><br>Secondary<br><br>Sliding percentage Subjective Symptoms of Stress and Well-Being at the Moment	general stress (F=6.46, p=.01), improvement in work-related well-being (F=12.1, P=.001)<br><br>Similarly, at post-intervention the intervention group produces significant decreases in perceived stress (F=33.4, P<.001), increases in WHO-5 scores (F=7.07, p<.001), greater improvement in work-related stress (F=5.50, p=.004), reduction in general stress (F=8.59, P<.001), and improvement in work-related well-being (F=8.92, p<.001)<br><br>only the intervention group presented a clinically significant change in the PSS-10 score (−6.15 points, on average) at

			T3: 8 weeks follow-up after randomisation (post -intervention)				the end of the 8-week study period, reaching symptom-free status (PSS-10=15.6),<br><br>After 8 weeks, the control group had a WHO-5 increase of approximately 6%, whereas improvement for the intervention group was almost 17%. Therefore, the difference in WHO-5 scores between groups was approximately 11%, which is clinically significant.
51	Wolever et al. (2012)	a national insurance carrier, USA	I: a mindfulness-based stress management intervention (2 versions-1 online virtual classroom that allowed for real-time bidirectional communication) teaches mindfulness practices that explicitly target work-related stress, work-life	Employees (n=239)<br><br>I(online): 52<br><br>I (in person mindfulness): 44<br><br>C: 53	RCT	Primary outcome:<br><br>PSS total Score<br><br>Secondary outcome:<br><br>The Center for Epidemiological Studies Depression Scale	No significant time x group interaction effects for the online intervention

			balance, and self-care. These practices are relatively brief (5–15 min) and are specifically designed to be used at work, 12 weekly hour-long classes, and a 2-hr mindfulness practice intensive at week 10.<br><br>I2: therapeutic yoga-based stress reduction program (in-person)<br><br>C: list of resources available to all employees of the national insurance carrier: discounted fitness programs, employee assistance programs, behavioural health services for depression, chair massage sessions, and wellness coaching opportunities.<br><br>Duration: 12 weeks (1 hr per week at the workplace, either in-person or online)<br><br>T2: post intervention			Cognitive and Affective Mindful-<br><br>ness Scale–Revised<br><br>Work Limitations Questionnaire	

<u>Section S1: Search Strategy</u>

MEDLINE ovid

((digital or online or on-line or internet-based or internet* or web-based or web* or computer-based or app* or mobile* or computer* or wearable*) and (psychological well-being or psychological well-being or resilience or stress* or occupational stress* or mental health or e-mental health or mental well-being or mental well-being or anxiety or depress* or burn-out or burnout or work engagement or work-engagement) and (intervention or stress prevention or stress management or problem solving or problem-solving or self-help or self help or CBT or mindfulness or mindfulness-based or cognitive behav* therapy)).ti. and (workplace or work or empl* or worker* or organis* or staff or job* or work-related or business).ti. and ENGLISH

OR

((digital or online or on-line or internet-based or internet* or web-based or web* or computer-based or app* or mobile* or computer* or wearable*) and (psychological well-being or psychological well-being or resilience or stress* or occupational stress* or mental health or E-mental health or mental well-being or mental well-being or anxiety or depress* or burn-out or burnout or work engagement or work-engagement) and (intervention or stress prevention or stress management or problem solving or problem-solving or self-help or self help or CBT or mindfulness or mindfulness-based or cognitive behav* therapy)).ab. and (workplace or work or empl* or worker* or organis* or staff or job* or work-related or business).ab. and ENGLISH

OR

((digital or online or on-line or internet-based or internet* or web-based or web* or computer-based or app* or mobile* or computer* or wearable*) and (psychological well-being or psychological well-being or resilience or stress* or occupational stress* or mental health or E-mental health or mental well-being or mental well-being or anxiety or depress* or burn-out or burnout or work engagement or work-engagement) and (intervention or stress prevention or stress management or problem solving or problem-solving or self-help or self help or CBT or mindfulness or mindfulness-based or cognitive behav* therapy)).kw. and (workplace or work or empl* or worker* or organis* or staff or job* or work-related or business).kw and JOURNAL ARTICLE

CINAHL

TI (("digital" or "online" or "on-line" or "internet-based" or "internet*" or "web-based" or "web*" or "computer-based" or "app*" or "mobile*" or "computer*" or "wearable*")) AND TI (("psychological well-being" or "psychological well-being" or "resilience" or "stress*" or occupational stress*" or "mental health" or "e-mental health" or "mental well-being" or "mental well-being" or "anxiety" or "depress*" or "burn-out" or "burnout" or "work engagement" or "work-engagement")) AND TI ("intervention" or "stress prevention" or "stress management" or "problem solving" or "problem-solving" or "self-help" or "self help" or "CBT" or "mindfulness" or "mindfulness-based" or "cognitive behav* therapy")) AND TI ("workplace" or "work*" or "empl*" or "worker*" or "organis*" or "staff*" or "job*" or "work-related" or "business"))

OR

"AB (("digital" or "online" or "on-line" or "internet-based" or "internet*" or "web-based" or "web*" or "computer-based" or "app*" or "mobile*" or "computer*" or "wearable*")) AND AB (("psychological well-being" or "psychological well-being" or "resilience" or "stress*" or occupational stress*" or "mental health" or "e-mental health" or "mental well-being" or "mental well-being" or "anxiety" or "depress*" or "burn-out" or "burnout" or "work engagement" or "work-engagement")) AND AB (("intervention" or "stress prevention" or "stress management" or "problem solving" or "problem-solving" or "self-help" or "self help" or "CBT" or "mindfulness" or "mindfulness-based" or "cognitive behav* therapy")) AND AB (("workplace" or "work*" or "empl*" or "worker*" or "organis*" or "staff*" or "job*" or "work-related" or "business))

OR

"SU ("digital" or "online" or "on-line" or "internet-based" or "internet*" or "web-based" or "web*" or "computer-based" or "app*" or "mobile*" or "computer*" or "wearable*") AND SU ("psychological well-being" or "psychological well-being" or "resilience" or "stress*" or occupational stress*" or "mental health" or "e-mental health" or "mental well-being" or "mental well-being" or "anxiety" or "depress*" or "burn-out" or "burnout" or "work engagement" or "work-engagement") AND SU ("intervention" or "stress prevention" or "stress management" or "problem solving" or "problem-solving" or "self-help" or "self help" or "CBT" or "mindfulness" or "mindfulness-based" or "cognitive behav* therapy") AND SU ("workplace" or "work*" or "empl*" or "worker*" or "organis*" or "staff*" or "job*" or "work-related" or "business"))

English

Peer reviewed

Psych info OVID

((digital or online or on-line or internet-based or internet* or web-based or web* or computer-based or app* or mobile* or computer* or wearable*) and (psychological well-being or psychological well-being or resilience or stress* or occupational stress* or mental health or E-mental health or mental well-being or mental well-being or anxiety or depress* or burn-out or burnout or work engagement or work-engagement) and (intervention or stress

prevention or stress management or problem solving or problem-solving or self-help or self help or CBT or mindfulness or mindfulness-based or cognitive behav* therapy) and (workplace or work or empl* or worker* or organis* or staff or job* or work-related or business)).ti. and Engl

OR

((digital or online or on-line or internet-based or internet* or web-based or web* or computer-based or app* or mobile* or computer* or wearable*) and (psychological well-being or psychological well-being or resilience or stress* or occupational stress* or mental health or E-mental health or mental well-being or mental well-being or anxiety or depress* or burn-out or burnout or work engagement or work-engagement) and (intervention or stress prevention or stress management or problem solving or problem-solving or self-help or self help or CBT or mindfulness or mindfulness-based or cognitive behav* therapy) and (workplace or work or empl* or worker* or organis* or staff or job* or work-related or business)).ab. and Engl

OR

((digital or online or on-line or internet-based or internet* or web-based or web* or computer-based or app* or mobile* or computer* or wearable*) and (psychological well-being or psychological well-being or resilience or stress* or occupational stress* or mental health or E-mental health or mental well-being or mental well-being or anxiety or depress* or burn-out or burnout or work engagement or work-engagement) and (intervention or stress prevention or stress management or problem solving or problem-solving or self-help or self help or CBT or mindfulness or mindfulness-based or cognitive behav* therapy) and (workplace or work or empl* or worker* or organis* or staff or job* or work-related or business)).id. and English

AND Peer-reviewed journal

EMBASE 0VID

((digital or online or on-line or internet-based or internet* or web-based or web* or computer-based or app* or mobile* or computer* or wearable) and (psychological well-being or psychological well-being or resilience or stress* or occupational stress* or mental health or E-mental health or mental well-being or mental well-being or anxiety or depress* or burn-out

or burnout or work engagement or work-engagement) and (intervention or stress prevention or stress management or problem solving or problem-solving or self-help or self help or CBT or mindfulness or mindfulness-based or cognitive behav* therapy) and (workplace or work or empl* or worker* or organis* or staff or job* or work-related or business)).ti. and English

OR

((digital or online or on-line or internet-based or internet* or web-based or web* or computer-based or app* or mobile* or computer* or wearable) and (psychological well-being or psychological well-being or resilience or stress* or occupational stress* or mental health or E-mental health or mental well-being or mental well-being or anxiety or depress* or burn-out or burnout or work engagement or work-engagement) and (intervention or stress prevention or stress management or problem solving or problem-solving or self-help or self help or CBT or mindfulness or mindfulness-based or cognitive behav* therapy) and (workplace or work or empl* or worker* or organis* or staff or job* or work-related or business)).ab. and English

OR

((digital or online or on-line or internet-based or internet* or web-based or web* or computer-based or app* or mobile* or computer* or wearable) and (psychological well-being or psychological well-being or resilience or stress* or occupational stress* or mental health or E-mental health or mental well-being or mental well-being or anxiety or depress* or burn-out or burnout or work engagement or work-engagement) and (intervention or stress prevention or stress management or problem solving or problem-solving or self-help or self help or CBT or mindfulness or mindfulness-based or cognitive behav* therapy) and (workplace or work or empl* or worker* or organis* or staff or job* or work-related or business)).kw. and English and Article, article in press, conference paper

Cochrane Central Register of Controlled Trials

Trials matching ((digital or online or on-line or internet-based or internet* or web-based or web* or computer-based or app* or mobile* or computer* or wearable*) and (psychological well-being or psychological well-being or resilience or stress* or occupational stress* or mental health or E-mental health or mental well-being or mental well-being or anxiety or depress* or burn-out or burnout or work engagement or work-engagement) and (intervention or stress prevention or stress management or problem solving or problem-solving or self-help

or self help or CBT or mindfulness or mindfulness-based or cognitive behav* therapy) and (workplace or work or empl* or worker* or organis* or staff or job* or work-related or business)) in Record Title OR ((digital or online or on-line or internet-based or internet* or web-based or web* or computer-based or app* or mobile* or computer* or wearable) and (psychological well-being or psychological well-being or resilience or stress* or occupational stress* or mental health or E-mental health or mental well-being or mental well-being or anxiety or depress* or burn-out or burnout or work engagement or work-engagement) and (intervention or stress prevention or stress management or problem solving or problemsolving or self-help or self help or CBT or mindfulness or mindfulness-based or cognitive behav* therapy) and (workplace or work or empl* or worker* or organis* or staff or job* or work-related or business)) in Abstract OR ((digital or online or on-line or internet-based or internet* or web-based or web* or computer-based or app* or mobile* or computer* or wearable) and (psychological well-being or psychological well-being or resilience or stress* or occupational stress* or mental health or E-mental health or mental well-being or mental well-being or anxiety or depress* or burn-out or burnout or work engagement or workengagement) and (intervention or stress prevention or stress management or problem solving or problem-solving or self-help or self help or CBT or mindfulness or mindfulness-based or cognitive behav* therapy) and (workplace or work or empl* or worker* or organis* or staff or job* or work-related or business)) in Keyword - (Word variations have been searched)

Web of science

(#2 OR #1) *AND* **LANGUAGE:** (English) *AND* **DOCUMENT TYPES:** (Article OR Proceedings Paper)

Refined by: DOCUMENT TYPES: (ARTICLE OR PROCEEDINGS PAPER OR EARLY ACCESS)

Indexes=SCI-EXPANDED, SSCI, AandHCI, CPCI-S, CPCI-SSH, ESCI, CCREXPANDED, IC Timespan=1995-2019

(#2 OR #1) *AND* **LANGUAGE:** (English) *AND* **DOCUMENT TYPES:** (Article OR Proceedings Paper)

Indexes=SCI-EXPANDED, SSCI, AandHCI, CPCI-S, CPCI-SSH, ESCI, CCREXPANDED, IC Timespan=1990-2019

(TS=((digital or online or on-line or internet-based or internet* or web-based or web* or computer-based or app* or mobile* or computer* or wearable) and (psychological well-being or psychological well-being or resilience or stress* or occupational stress* or mental health or E-mental health or mental well-being or mental well-being or anxiety or depress* or burn-out or burnout or work engagement or work-engagement) and (intervention or stress prevention or stress management or problem solving or problemsolving or self-help or self help or CBT or mindfulness or mindfulness-based or cognitive behav* therapy) and (workplace or work or empl* or worker* or organis* or staff or job* or work-related or business))) *AND* **LANGUAGE:** (English) *AND* **DOCUMENT TYPES:** (Article OR Proceedings Paper)

Indexes=SCI-EXPANDED, SSCI, AandHCI, CPCI-S, CPCI-SSH, ESCI, CCREXPANDED, IC Timespan=1990-2019

(TI=((digital or online or on-line or internet-based or internet* or web-based or web* or computer-based or app* or mobile* or computer* or wearable) and (psychological well-being or psychological well-being or resilience or stress* or occupational stress* or mental health or E-mental health or mental well-being or mental well-being or anxiety or depress* or burn-out or burnout or work engagement or work-engagement) and (intervention or stress prevention or stress management or problem solving or problemsolving or self-help or self help or CBT or mindfulness or mindfulness-based or cognitive behav* therapy) and (workplace or work or empl* or worker* or organis* or staff or job* or work-related or business))) *AND* **LANGUAGE:** (English) *AND* **DOCUMENT TYPES:** (Article OR Proceedings Paper)

Indexes=SCI-EXPANDED, SSCI, AandHCI, CPCI-S, CPCI-SSH, ESCI, CCREXPANDED, IC Timespan=1990-2019

Section S2: Evidence Map

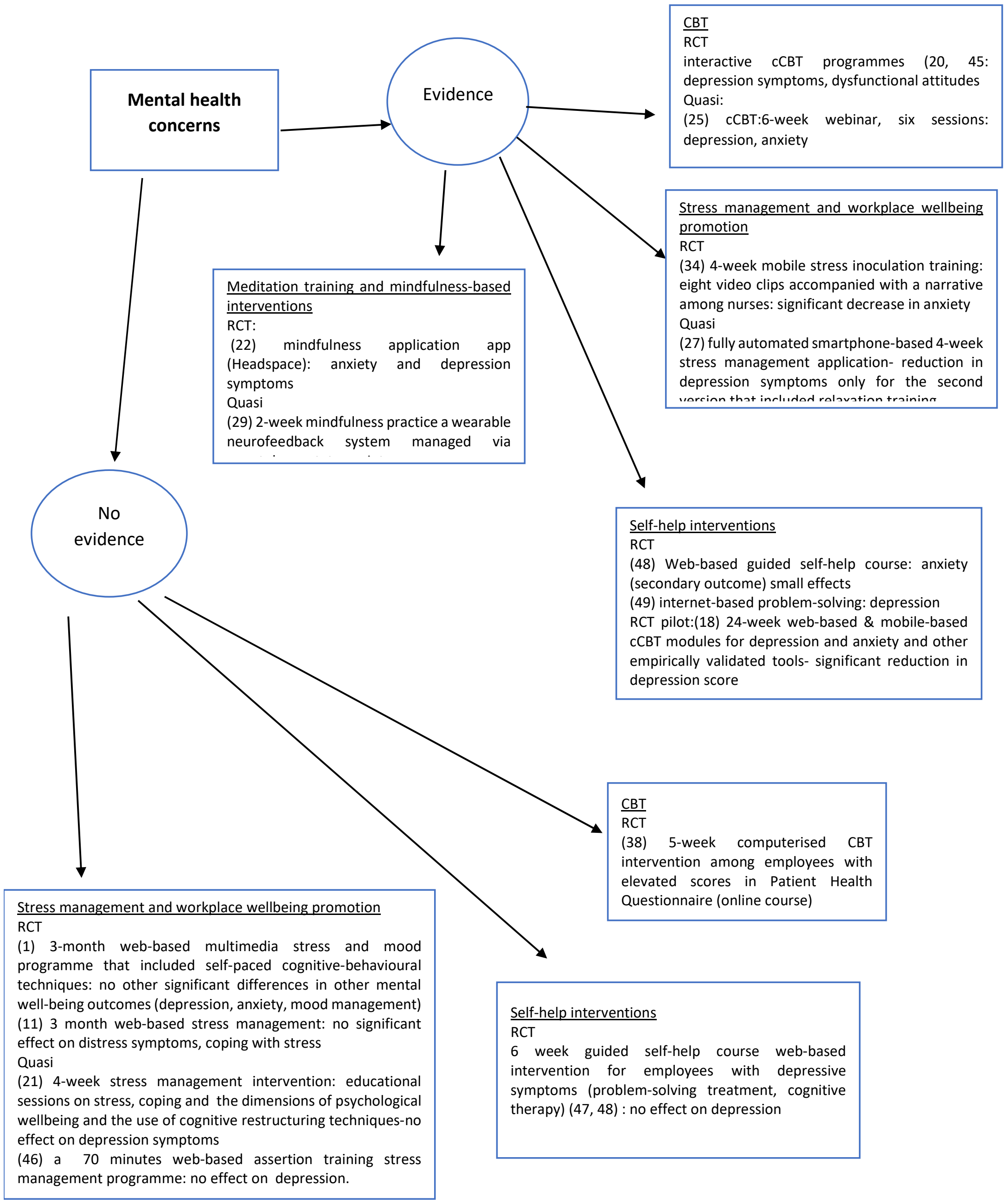

Work-related wellbeing

Evidence

CBT
RCT
(20) interactive cCBT programme marginally significant effect on distress
(26): marginally significant indirect effects on work-engagement through changes in depression (p's<0.1)

Meditation training and mindfulness-based interventions
RCT
(22) mindfulness application app (Headspace): job strain & and workplace support
7-week mindfulness training (33) adaptation of mindfulness-based stress reduction (a moderated discussion board) significant small to medium effect & at 3-month follow-up but no effects on mindfulness.
(40) 4-week instructor-led (through online instructional videos) mindfulness training that combined elements of mindfulness-based stress reduction & mindfulness-based cognitive therapy: large effect on chronic and acute fatigue.
Quasi:
(24) 8-week online MBSR significant improvement for state anxiety & fatigue
(35) Headspace app once a week for 4 weeks marginally improved burnout and compassion satisfaction(and improved compassion fatigue for those with low levels of PTSD symptoms
(4, 29) 2-week mindfulness practice a wearable neurofeedback system managed via smartphone (29) & 6-week yoga therapy meditation program delivered and tracked by a smartphone app (4) significant improvement for burnout (4), mindfulness (4) (perceived stress (29), fatigue scores (29) and compassion fatigue (4)
(3) online classes through six 50-minute interactive facilitated sessions of mantra repetition: beneficial effects in the frequency of Stressful Events, troubled conscience about stressful events& a significant decline in feelings of exhaustion).

Self-help interventions
RCT
(10, 49):3-6 weeks evidence-based web-based cognitive & behavioural training (self-regulation, problem-solving): feedback, internet--based: overall stress, perceived stress, work-engagement
(48) Web-based guided self-help course (problem solving & cognitive therapy): emotional exhaustion (secondary outcome) small effects
RCT (pilot)
(28) online happiness training: stress warning, and particularly emotional stress
Quasi:
(7) one's month use mobile mental wellness-training app (ACT processes) significant improvement in (single-item) ratings of stress
(30) one's month use of a resilience mobile application (Provider Resilience app): significant improvement in burnout and compassion fatigue, no significant effect on compassion satisfaction, symptom distress, interpersonal relations and social role
(36) feasibility study-a web-based version of a previously standardised group intervention-behavioral intervention: perceived stress

Stress management and workplace wellbeing promotion
RCT's
(1) 3-month web-based multimedia health promotion programme that included self-paced cognitive-behavioural techniques tailored to individuals based on their earlier screening assessment. Only marginally significant reduction in stress
(2) self-help e-mental health approach: five online interventions tailored to participants' earlier screening work-functioning results. Significant improvement in work-related fatigue & work-functioning but not significant effects on stress
(11) 3-month web-based stress management created specifically for nurses 8 stress management modules: nurses' stress improvement
(12) videoconferencing-based telepsychology: emotional labour
(23) 7 weeks psychoeducation and 2-phased approach (skill acquisition & practice): marginally coping skills
43) 6-week smartphone administered stress intervention (based on ACT): significant medium effect-size for distress, significant medium effect-size for perceived stress
(32) cognitive-behavioral (4 modules, 2 sessions)-perceived stress (short-term
(37) 6-month web-based stress management and health promotion tool: perceived ability to manage stress, perceived social support
(50) 8-week app developed to promote stress management and well-being: significant decreases in perceived stress, general stress.
Quasi.
27) two versions of a fully automated smartphone-based 4-week stress management application Both personalized recommendations, psychoeducation and cognitive-behavioural techniques, the second version also included relaxation training: significant reduction in psychosocial stress
(46)70 minutes web-based assertion training stress management: improved significantly mental workload

No Evidence

Self-help
RCT
(9) micro-tasks
Quasi
(6) psychoeducation-no effects
30) one's month use of a resilience mobile application (Provider Resilience app): no significant effect on compassion satisfaction

Meditation training and mindfulness-based interventions
RCT
(8) 3-week mindfulness meditation practices via an app: no effect on perceived stress
(51) 12-week real-time long-hour virtual classes: no effect on perceived stress

Stress management & workplace wellbeing promotion
RCT
(12)videoconferencing-based telepsychology : no perceived stress
(23) 7 weeks psychoeducation & 2-phased approach (skill acquisition & practice): job stress & work-engagement
(32) cognitive-behavioral (4 modules, 2 sessions)-no long term effect in stress & in-person group better

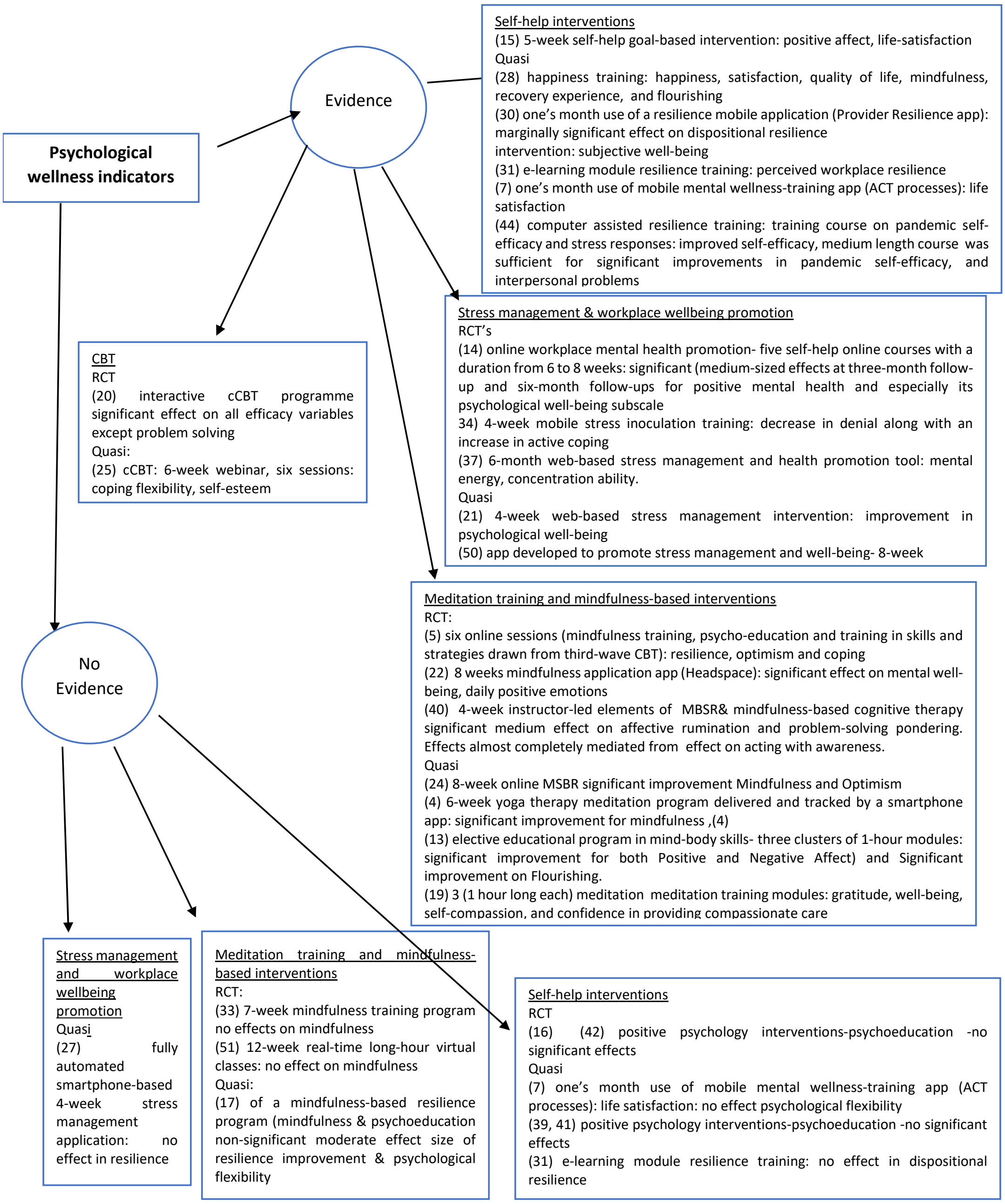
Psychological wellness indicators
Evidence
Self-help interventions
(15) 5-week self-help goal-based intervention: positive affect, life-satisfaction
Quasi
(28) happiness training: happiness, satisfaction, quality of life, mindfulness, recovery experience, and flourishing
(30) one's month use of a resilience mobile application (Provider Resilience app): marginally significant effect on dispositional resilience
intervention: subjective well-being
(31) e-learning module resilience training: perceived workplace resilience
(7) one's month use of mobile mental wellness-training app (ACT processes): life satisfaction
(44) computer assisted resilience training: training course on pandemic self-efficacy and stress responses: improved self-efficacy, medium length course was sufficient for significant improvements in pandemic self-efficacy, and interpersonal problems
Stress management & workplace wellbeing promotion
RCT's
(14) online workplace mental health promotion- five self-help online courses with a duration from 6 to 8 weeks: significant (medium-sized effects at three-month follow-up and six-month follow-ups for positive mental health and especially its psychological well-being subscale
34) 4-week mobile stress inoculation training: decrease in denial along with an increase in active coping
(37) 6-month web-based stress management and health promotion tool: mental energy, concentration ability.
Quasi
(21) 4-week web-based stress management intervention: improvement in psychological well-being
(50) app developed to promote stress management and well-being- 8-week
CBT
RCT
(20) interactive cCBT programme significant effect on all efficacy variables except problem solving
Quasi:
(25) cCBT: 6-week webinar, six sessions: coping flexibility, self-esteem
Meditation training and mindfulness-based interventions
RCT:
(5) six online sessions (mindfulness training, psycho-education and training in skills and strategies drawn from third-wave CBT): resilience, optimism and coping
(22) 8 weeks mindfulness application app (Headspace): significant effect on mental well-being, daily positive emotions
(40) 4-week instructor-led elements of MBSR& mindfulness-based cognitive therapy significant medium effect on affective rumination and problem-solving pondering. Effects almost completely mediated from effect on acting with awareness.
Quasi
(24) 8-week online MSBR significant improvement Mindfulness and Optimism
(4) 6-week yoga therapy meditation program delivered and tracked by a smartphone app: significant improvement for mindfulness ,(4)
(13) elective educational program in mind-body skills- three clusters of 1-hour modules: significant improvement for both Positive and Negative Affect) and Significant improvement on Flourishing.
(19) 3 (1 hour long each) meditation meditation training modules: gratitude, well-being, self-compassion, and confidence in providing compassionate care
No Evidence
Stress management and workplace wellbeing promotion
Quasi
(27) fully automated smartphone-based 4-week stress management application: no effect in resilience
Meditation training and mindfulness-based interventions
RCT:
(33) 7-week mindfulness training program no effects on mindfulness
(51) 12-week real-time long-hour virtual classes: no effect on mindfulness
Quasi:
(17) of a mindfulness-based resilience program (mindfulness & psychoeducation non-significant moderate effect size of resilience improvement & psychological flexibility
Self-help interventions
RCT
(16) (42) positive psychology interventions-psychoeducation -no significant effects
Quasi
(7) one's month use of mobile mental wellness-training app (ACT processes): life satisfaction: no effect psychological flexibility
(39, 41) positive psychology interventions-psychoeducation -no significant effects
(31) e-learning module resilience training: no effect in dispositional resilience

JBI Data Extraction Form for Experimental/Observational Studies

Reviewer ______________________________ Date ________________

Author ______________________________ Year ________________

Journal _ Record Number ________

Study Method RCT ☐ Quasi-RCT ☐ Longitudinal ☐

Retrospective ☐ Observational ☐ Other ☐

Participants

Setting: ____

Population: ____

Sample size:

Intervention 1__________ Intervention 2__________ Intervention 3 ________

Interventions

Intervention 1:____

Intervention 2:____

____

Intervention 3: ____

____

Clinical outcome measures

Outcome Description	Scale/measure

Study results

Dichotomous data

Outcome	Intervention ( ) number / total number	Intervention ( ) number / total number

Outcome	Intervention (x) mean and SD	Control mean and SD

Comments:

Section S3: Full theory-base assessment

Study id	Theory constructs: Are specific models/theories explicitly mentioned and their relationship with targeted psychological constructs?	Intervention components: Are intervention techniques explicitly linked to theory relevant constructs?	Significant effects in favour of the intervention.
1	no	No<br><br>No links with theory-relevant constructs	partial
5	Partial<br>Not specific theory mentioned<br><br>Different resilience measures<br>“We utilized both measures at baseline and 6-month follow-up in our study to examine 2 inherent constructs of resilience: (1) successful adaptation to stressful life events and circumstances and (2) bounce-back resilience”	no but evidence-based<br><br>mindfulness training may serve to enhance psychological resilience is further supported by findings from a recent meta-analysis that found resilience can indeed be enhanced, particularly by programs that involve both cognitive behavioural strategies and mindfulness training”	yes
8	no	Partial<br>“The hypothesized mechanism by which meditation has this effect is by inducing a feeling of relaxation, which decreases physiological arousal”	No
9	no	No: intervention components not based on specific theoretical model<br><br>“micro-tasks were specifically crafted for physicians and intentionally designed to cultivate professional satisfaction and well-being in one of 6 domains:<br><br>• Promote meaning in work and job satisfaction, • Foster teamwork and social support at work, • Nurture personal relationships and work-life balance, • Recognize and build on personal strengths (courage, honesty, patience, wisdom, humanity, justice, and transcendence),<br><br>• Encourage effective problem solving, and • Promote positive emotions.<br><br>These 6 themes were informed by a robust literature on<br><br>physician career satisfaction, well-being, positive psychology, and mindfulness (Brunwasser, Gillham, and Kim, 2009; Krasner et al., 2009; Lyubomirksy, 2008; Shapria and Mongrain, 2010).”	no
10	No	Yes<br><br>imagery procedure based on the integration of two established self-regulation strategies: mental contrasting and forming implementation intention)	Partially yes

11	no	No<br><br><br>No links with theory-relevant constructs	partial
12	No	no	no
14	Partial: theory-based constructs but not one specific theoretical model<br><br>a dynamic concept of health to justify specific psychological well-being measures (positive mental health, subjective well-being measures)<br><br>“well-being acts as a protective factor for mental health and healthy functioning. Three different types of well-being have been identified in various studies. Subjective well-being refers to positive affect and/ or life satisfaction (Diener, 1984). Psychological well-being refers to the level of positive functioning containing constructs such as meaning in life, goal setting, and mastery (Ryff, 1989). Social well-being contains constructs such as the level of social integration and social contribution, together forming the construct of ‘positive mental health’ (Keyes, 2002).”	Yes: “From self-determination theory it can be reasoned that the opportunity to choose an intervention that fits the preference and need of its user would promote autonomy”	yes
15	Partial: theory-based constructs but not one specific theoretical model<br><br><br>broader mental well-being concept to justify specific psychological well-being measure (flourishing measure)	Yes: Module 1 asked participants to set personal goals (in work or home life) and refine them to ensure they were personally important to them (self-concordant), not determined by others. Working towards voluntary, self-concordant goals can improve PA (Lyubomirsky et al., 2005) and life satisfaction (Judge, Bono, Erez, and Locke, 2005). In Module 2, participants were asked to imagine achieving their goals, which can improve motivation to actively pursue goals (Oettingen,Mayer,and Brinkmann, 2010) and increase an individual’s sense ofpurpose, one aspect of flourishing (Schmitt, Zacher, and de Lange, 2013). Module 3 encouraged participants to make written plans for achieving their goals. Having specific and realistic plans to achieve goals brings improvements in PA, life satisfaction, and flourishing, even before the goals have actually been achieved (Cheavens, Feldman, Gum, Michael, and Snyder, 2006; MacLeod and Conway, 2005; Prenda and Lachman, 2001). Modules 4 and 5	yes

		involved participants putting their plans into action, andmaking amendments to goals and to plans in response to obstacles. Discarding unattainable goals can reduce NA, while pursuing revised goals can increase PA and a sense ofpurpose in life (Wrosch, Scheier, and Miller, 2013).	
16	no;<br><br>“Well-being can be defined in terms of feeling good: balanced positive and negative affect and satisfaction with life, and doing well: a positive perception of optimal functioning of the individual and in the society”.<br><br>Referencing many theoretical principles of positive psychology but not measuring of theory-based targeted constructs or adopting one clear theory	no	no
18	no	No but evidence-based<br><br>No links with theory-relevant constructs	yes
20	yes:<br><br>cognitive restructuring was adopted for targeting and measuring dysfunctional attitude	Yes<br><br>cognitive restructuring skills development as the study’s main cognitive approach	Yes
22	-Yes:<br>Authors reference Karasek’s model (1979) of job-demand control model that define psychosocial stress at work justifying their choice of focusing on the construct of job strain and measuring a range of psychosocial outcomes: Job strain (16 items extracted from the Whitehall II Study Questionnaire), workplace Social support at work (five statements ranked on a 4-point scale)	Yes<br>meditations are in line with a two-component model of mindfulness, for which the first component is the regulation of attention in order to focus it on the present moment (e.g., through paying attention to the breath), and the second component is open monitoring in which thoughts and emotions that arise are treated with curiosity, openness, and acceptance (Bishop et al., 2004). (study id: 22)<br><br>-Workplace social support effects: “supports theoretical perspectives of mindfulness suggesting that in addition to improving an individual’s psychological well-being, practicing mindfulness improves one’s relationship with others (Brown, Ryan, and Creswell, 2007).” (study id: 22)	yes
23	No<br><br><br>Referencing stress management and positive psychology literature without adopting specific theoretical models	Partial : According to social learning theory, self-efficacy beliefs (judgments regarding one’s capabilities) and outcome expectancies (judgments regarding the consequences of behaviours) significantly influence coping behaviours23). The goal of the skill acquisition phase was to increase participants’ ‘outcome expectancies’ about intervention related outcomes, and the goal of the practice phase was to increase ‘efficacy expectancy’	no

26	no	yes<br>cognitive restructuring skills development as the study's main cognitive approach	Partial (significant small effect for work-engagemen t)
28	Partial:<br>Lyubomirsky et al. [8] approach on happiness but no specific links with outcome measures or theory-based relationships<br>"a person's happiness level is determined by 3 factors: a genetic set point for happiness, happiness-relevant circumstances, and happiness-related activities and practices. Furthermore, the authors found that happiness-related Because the brain, principally, never loses its ability to adapt [9], happiness (and, along with it, well-being) is learnable at almost every age"<br>Hypothesis:<br>happiness training improves individual happiness and satisfaction with life and relieves stress, and, as a consequence thereof, reduces stress-related symptoms in an occupational setting	Yes: Positive psychology interventions "treatment methods or intentional activities aimed at cultivating positive feelings, positive behaviours, or positive cognitions"	yes
32	no	no	no
33	no but evidence-base informed	no but evidence-base informed	partial
34	Yes<br>(Karasek job strain model)-job content questionnaire<br>"Considering the kind of job and patients' features, data were strictly related to the low perception of autonomous decision making at work"	Yes<br>Stress inoculation training<br>(conceptualisation phase, skills acquisition phase, application and follow-through phase)<br>"The skills acquisition and rehearsal phase was combined with two kinds of relaxation techniques: the Progressive Muscular Relaxation (PMR; Jacobson, 1938) and Autogenic Training (AT; Schultz, 1977). PMR (Jacobson, 1938) aims to decrease the physiological aspects of anxiety by distracting individuals from their awareness of anxious feelings. AT (Schultz, 1977) explores the effectiveness of a relaxation training based on the individual's ability to control the body through mind exercises."	yes

37	no	No<br><br>No links with theory-relevant constructs	partial
38	no	No<br><br>No links with theory-relevant constructs	no
40	Yes:<br>Mindfulness mechanisms of change: “Shapiro, Carlson, Astin, and Freedman (2006) suggest two approaches to assessing mechanisms of change in mindfulnessbased intervention” affective rumination and problem solving pondering and measures	no but evidence based	yes
42	No<br><br>Referencing optimising well-being literature for the promotion of the development of psychological resources<br><br>but not measuring of theory-based targeted constructs or adopting one clear theory	yes: ROL enhances resilience by teaching users seven core skills of resilience, based on cognitive therapy. The user is guided through the skills by a video of a psychologist and accompanying slides. The program enables users to interact at any time during the program with several Virtual Partners that help users understand the key learning components from multiple perspectives; table seven components of resilience: emotion regulation, impulse control, optimism, causal analysis, empathy, self-efficacy and reaching out; graphical feedback is provided on resilience strengths and areas for improvement, along with a table indicating which resilience skill will help improve each area of resilience, The user then makes an action plan by identifies how he/she can apply some of the skills to everyday life	no
43	Partial: “parallels between the presumed mechanisms in ACT and the theory of transformative leadership” and leadership effectiveness measure: Multifactor Leadership<br><br>Questionnaire (MLQ)	Partial:<br><br>Intervention components teaching ACT principles without linking each intervention component with theory-relevant constructs	partial
45	Targeting attributional style measured by The Attributional Style Questionnaire (ASQ) that refers to the optimism or pessimism with which people explain why things happen to them [and is inversely associated with depression	Yes<br><br>Its cognitive components explore automatic thoughts, thinking errors and distraction, challenging unhelpful thinking, core beliefs and attributional style, The behavioural components of ‘Beating The Blues’ for tackling specific problems comprise: activity scheduling, task breakdown, problem solving, sleep management, relaxation training and biofeedback, planning and prioritizing and graded exposure	Partial (only up to 1 month)
47	no	No<br><br>No links with theory-relevant constructs	no

48	no	Yes<br><br>"depressive symptoms can be caused by practical problems that people face in their daily lives. It is believed that when people can resolve their problems, their symptoms of depression will decrease [38]. The PST will help them solve their problems. Sometimes, however, problem solving can be disrupted by automatic thoughts"… Therefore, we incorporated cognitive therapy information and assignments to change these automatic thoughts in the course"	Partial
49	Yes<br><br>(problem solving therapy and ineffective coping behaviour, and adverse effects)<br><br>Coping skills measure and job content questionnaire	Partially: " Guidance was conceptualized according to a theoretical model for providing guidance in eHealth interventions"<br><br>No links with theory-relevant constructs	Partially
50	Yes:<br><br>mechanisms actions of mindfulness and positive mental health)-<br><br>relaxation response and mental processes	No but evidence-based:	yes
51	no	Partial<br>"Relaxation component of the intervention decreasing physical arousal."	No

Section S4: JBI assessment-both reviewers

Question 1 Is it clear in the study what is the 'cause' and what is the 'effect' (i.e., there is no confusion about which variable comes first)?

13, 19, 27, 30, 31, 36-?

I rated those questions "Yes" because they all include an intervention and seek to assess its effect.

Question 2: Were the participants included in any comparisons similar?

19: I rated it "Unclear" because it mentions that "Data were collected from 3 online meditation training modules" from May 2014 to October 2015 and then it offers description of participant characteristics that completed at least one (not participant characteristics in each of the modules) while participant details were collected upon registration.

36: I rated it "Yes" because before and after measures are reported for the same one group, and there is also a graph showing the differences between "officer" and "enlisted"

Question 3: Were the participants included in any comparisons receiving similar treatment/care, other than the exposure or intervention of interest?

2: I rated it "Yes" because they the waiting-list group of a previous randomized controlled trial

6: I rated it "Unclear" only because one of the limitations the authors mention is that participants in both groups were working together and that some in the control group mentioned that this intervention was "a topic of conversation in the waiting room"

27:I rated it "No" because the intervention in 2014 included relaxation training

35:I rated it "No" because there were two single group samples for two different interventions

41: I rated it "Yes" because it mentions that "*the process of participating in the interventions did not start for the wait list control group until the activity period had ended for the other two groups*"

Question 5: Were there multiple measurement of the outcome both pre- and post- the intervention/exposure?

13: I rated it "No" because some measures were measured before only

19: I rated it "Yes" because the same pre- and post- measures were collected.

31: I rated "No" because it is post-test only design

Question 6: (Shall we rate studies that were not designed to have a follow-up as "No" or "N/A"?) Was follow up complete and if not, were differences between groups in terms of their follow-up adequately described and analysed?

21: I rated it "No" because although it involves a process evaluation the overall design is pre-post without follow-up.

25: I rated it “Yes” because data were collecter pre (T_0), post (T_1), and 1 month after (T_2) the intervention

27: I rated it “No” because it involve two pre-post single groups (one pre-post in 2013 and another pre-post group in 2014)

41: I rated it “No” because it reports pre-post measures for two groups and a control one.

Question 8: Were the outcomes measured in a reliable way?

4: I rated it “yes” because it mentions the Cronbach alpha’s for the PROQOL and MAIA scale for this study

27: I rated it “Unclear” because it mentions that the scales had shown previously good reliability, but I couldn’t find anything about this specific study.

31: (Yes or Unclear?) I rated it “yes” because it mentions that “*Reliabilities, assessed by combining data across all samples,* “

Question 9: Was appropriate statistical analysis used?

13: I rated it “yes” because the sample was large (also intervention program was offered for free, was voluntary, and without university credits)

19: I rated it “yes” because paired t-tests were conducted with a large sample

24: I rated it “Unclear” because t-test were used with a small sample, and Low power but on the other hand it included of physiological measures as an outcome measure

25: I rated it “No” because they used Linear Mixed Effects Modelling but with small sample, low power and high attrition

36: I rated it “Yes” because they used paired t-test with a large sample
